# TMS coil orientation: from computational modeling to experimental evidence and analgesic considerations

**DOI:** 10.3389/fnins.2026.1938946

**Published:** 2026-10-19

**Authors:** Armina Saadatkhah, Albert Leung

**Affiliations:** 1 University of California, San Diego, San Diego, CA, United States; 2 Department of Anesthesiology, Veteran Affairs San Diego Healthcare System, Center for Pain and Headache Research, The University of California, San Diego, San Diego, CA, United States

**Keywords:** coil orientation, electric field modeling, motor cortex, neuromodulation, transcranial magnetic stimulation

## Abstract

**Introduction:**

Transcranial magnetic stimulation (TMS) is a widely used non-invasive brain stimulation technique whose physiological and clinical effects for pain-related indications may be critically shaped by the orientation of the stimulating coil. This scoping review aims to: 1) examine optimal coil orientation for the primary motor cortex (M1), with particular attention to the hand representation, and the dorsolateral prefrontal cortex (DLPFC), as predicted by computational modeling; 2) evaluate orientation-dependent physiological effects in healthy subjects at both M1 & DLPFC; and 3) assess evidence for coil orientation specific analgesic effects of repetitive TMS (rTMS) at M1 or DLPFC.

**Methods:**

Following the PRISMA-ScR guidelines, seven databases (PubMed, Embase, Web of Science, Cochrane Library, ScienceDirect, DOAJ, IEEE Xplore) were searched for studies from 1992 to 2025 that compared two or more figure-of-eight coil orientations, or derived an optimal orientation, at M1 or the DLPFC. Risk of bias in the 18 studies involving human participants was assessed using ROBINS-I V2.

**Results:**

Twenty-four M1-related studies (8 computational, 16 experimental) and three DLPFC-related studies (2 experimental, 1 mixed experimental & computational) met the eligibility criteria. Three retrieved review articles were retained only as secondary sources. Review of the computational modeling articles indicates that while the 45° posterior-to-anterior (PA45°) orientation, referenced to the interhemispheric longitudinal fissure, provides a reliable and physiologically justified angle for M1 hand-area stimulation, optimal orientation is determined by local cortical geometry rather than a fixed angle. Computational models consistently demonstrate that the optimal angle, which induces the strongest electric field (E-field), is perpendicular to the local sulcal wall and parallel to the principal axis of the underlying cortical columns, corresponding to approximately PA45° for the M1 hand area, but differing for lower-limb representations, the pre-supplementary motor area, and the DLPFC. Human experimental findings are consistent with these predictions at M1, whereas DLPFC evidence remains sparse to specify an optimal angle. No study compared coil orientations for therapeutic analgesic outcomes.

**Discussion:**

Coil orientation has been shown to influence the induced E-field and potentially the intended analgesic benefit. However, the latter remains uninvestigated. This gap is the review’s principal finding and defines a specific, testable research question.

## Introduction

Transcranial magnetic stimulation (TMS) non-invasively stimulates the brain by utilizing electromagnetic principles to produce focal neuronal events in the cortex (Wassermann, 1998; Wassermann and Lisanby, 2001). The device usually consists of an insulated electric coil, which, with the passing of an electrical current, generates a dynamic magnetic field through the scalp and skull, and into the first few millimeters of the cortex without attenuation. A figure-of-eight (FOE) TMS coil is commonly used clinically because it provides a more precise cortical localization compared to other coil designs, achieving depolarization of corticospinal tracts via the projected electric field (E-field) at approximately the junction of the grey and white matter (Epstein et al., 1990; Siebner et al., 2009). Due to this effect, TMS can function as a reversible “virtual lesion,” allowing researchers and clinicians to temporarily interfere with, or modulate local neural circuits (Gomez et al., 2021). Clinically, TMS is widely used to treat major depressive and other mood disorders. Increasingly, TMS has been applied to pain management, supported by growing clinical evidence (Leung et al., 2020). As these clinical applications expand, optimizing stimulation parameters becomes essential for improving both therapeutic effectiveness and reproducibility. Among the parameters that govern TMS outcomes, coil orientation is particularly significant because it can potentially determine the direction and spatial distribution of the induced E-field relative to cortical anatomy (Siebner et al., 2009; Deng et al., 2013) and downstream electrophysiological response, and ultimately, behavioral modulation. While extensive studies have assessed the effects of TMS coil orientation using computer-generated E-field models and human experimental paradigms, the influence of coil orientation on pain outcomes has not been comprehensively reviewed. Therefore, the current review aims to: (1) examine optimal coil orientation for the primary motor cortex (M1), with particular attention to the hand representation, and the dorsolateral prefrontal cortex (DLPFC), as predicted by computational modeling; (2) evaluate correlated orientation-dependent physiological effects in healthy subjects at both M1 & DLPFC; and (3) assess evidence for coil orientation specific analgesic effects of repetitive TMS (rTMS) at M1 or DLPFC.

## Methods

This article is reported as a scoping review, following the Preferred Reporting Items for Systematic Reviews and Meta-Analyses extension for Scoping Reviews [PRISMA-ScR (Tricco et al., 2018)]. A scoping review design was selected because the objective of this review was to map the extent, range, and nature of evidence on coil orientation across three heterogeneous literatures: computational dosimetry, human neurophysiology, and clinical pain trials, and to identify gaps, rather than to pool effect estimates for a single well-defined intervention–outcome pair. The heterogeneity of outcomes across these literatures (E-field magnitude, motor threshold, motor evoked potential (MEP) amplitude, hemodynamic response, pain threshold) precludes quantitative synthesis.

The review question was framed using the Population–Concept–Context structure recommended for scoping reviews:

*Population*: The specific population in this review is represented by healthy adult volunteers and adult patients undergoing TMS, together with individualized computational head models derived from human anatomical imaging.
*Concept*: The main concept examined is coil orientation, defined as the angular position of the FoE coil relative to a specified anatomical reference axis, and its relationship to induced E-field distribution, cortical excitability, and pain-related outcomes.
*Context*: The context this review draws from is the stimulation of the M1 or the DLPFC, in laboratory or clinical settings.

Expressed in the PICOS terms requested for interventional reviews, the question is: in adults receiving figure-of-eight TMS (P), does one coil orientation (I), compared with an alternative orientation at the same cortical target (C), alter induced E-field distribution, corticospinal excitability, or pain-related outcomes (O), across computational modeling studies, human experimental studies, and clinical trials (S)?

A comprehensive literature search was conducted across seven databases: PubMed, ScienceDirect, Directory of Open Access Journals (DOAJ), Web of Science, Cochrane Library, Embase, and IEEE Xplore. IEEE Xplore was searched in addition to the biomedical databases because the computational and dosimetry literature on TMS is published partly in engineering venues. It returned no records beyond those already retrieved by the other databases and therefore contributed no unique studies to this review. The final search was completed on February 2026. No language restrictions and no filters for study design, publication type, or age group were applied. Results were restricted to publications from 1992 to 2025; 1992 was selected because it is the year of the earliest study identifying coil orientation as a determinant of MEP amplitude, and no relevant earlier record was identified. One publication dated 2026 and one other dated 2025 were identified after the final search date through citation tracking and are cited as contextual or methodological references only; they were not screened, are not included in the study counts, and do not contribute to any synthesis in this review. Complete search strings for every database, including Boolean operators and field tags, are provided in Table 1 for the M1 search and Table 2 for the DLPFC search.

**Table 1:** Complete search strategy for the primary motor cortex (M1) literature search.

Database	Search string
PubMed	(“TMS coil orientation” OR “Transcranial Magnetic Stimulation coil orientation” OR “Transcranial Magnetic Stimulation 45°”) AND (“motor cortex” OR “M1”)
Embase	(‘transcranial magnetic stimulation’/exp. OR ‘TMS’) AND (‘coil orientation’ OR ‘45 degree*’) AND (‘motor cortex’/exp. OR ‘M1’)
Web of Science	TS = (“TMS coil orientation” OR “Transcranial Magnetic Stimulation coil orientation” OR “Transcranial Magnetic Stimulation 45°”)
Cochrane Library	(“TMS coil orientation” OR “Transcranial Magnetic Stimulation coil orientation”) in Title Abstract Keyword
ScienceDirect	“TMS coil orientation” OR “Transcranial Magnetic Stimulation coil orientation” (Title, abstract, keywords)
DOAJ	“TMS coil orientation” OR “coil orientation” AND “transcranial magnetic stimulation”
IEEE Xplore	(“transcranial magnetic stimulation” OR “TMS”) AND (“coil orientation” OR “coil angle” OR “current direction”) AND (“motor cortex” OR “M1” OR “motor evoked potential”)

Full Boolean search strings, including operators and field tags, are reported for each of the seven databases searched (PubMed, ScienceDirect, DOAJ, Web of Science, Cochrane Library, Embase, and IEEE Xplore), together with the records retrieved from each. Results were restricted to publications from 1992 to 2025. No language restrictions and no filters for study design, publication type, or age group were applied.

DOAJ, Directory of Open Access Journals; M1, primary motor cortex; TMS, transcranial magnetic stimulation.

**Table 2:** Complete search strategy for the dorsolateral prefrontal cortex (DLPFC) literature search.

Database	Search string
PubMed	(“TMS DLPFC coil orientation” OR “DLPFC coil orientation” OR “Dorsolateral Prefrontal Cortex coil orientation”)
Embase	(‘transcranial magnetic stimulation’/exp. OR ‘TMS’) AND (‘dorsolateral prefrontal cortex’/exp. OR ‘DLPFC’) AND (‘coil orientation’ OR ‘45 degree*’)
Web of Science	TS = (“TMS DLPFC coil orientation” OR “DLPFC coil orientation” OR “Dorsolateral Prefrontal Cortex coil orientation”)
Cochrane Library	(“DLPFC coil orientation” OR “Dorsolateral Prefrontal Cortex coil orientation”) in Title Abstract Keyword
ScienceDirect	“TMS DLPFC coil orientation” OR “DLPFC coil orientation” OR “Dorsolateral Prefrontal Cortex coil orientation” (Title, abstract, keywords)
DOAJ	“DLPFC coil orientation” OR “Dorsolateral Prefrontal Cortex coil orientation”
IEEE Xplore	(“transcranial magnetic stimulation” OR “TMS”) AND (“coil orientation” OR “coil angle” OR “current direction”) AND (“dorsolateral prefrontal cortex” OR “DLPFC” OR “prefrontal”)

Full Boolean search strings, including operators and field tags, are reported for each of the seven databases searched, together with the records retrieved from each. The M1 and DLPFC keyword sets are reported separately because the two searches were run independently. Restrictions and the final search date are as reported for Table 1.

DLPFC, dorsolateral prefrontal cortex; DOAJ, Directory of Open Access Journals; TMS, transcranial magnetic stimulation.

Motor cortex searches were conducted using combinations of the keywords “TMS coil orientation,” “Transcranial Magnetic Stimulation coil orientation,” and “Transcranial Magnetic Stimulation 45°.” A parallel search targeting DLPFC coil orientation was conducted with keywords “TMS DLPFC coil orientation,” “DLPFC coil orientation,” and “Dorsolateral Prefrontal Cortex coil orientation.” Records were included if they: (a) used a FoE coil or a functionally equivalent focal coil; (b) evaluated coil orientation as an independent variable, meaning that two or more orientations were compared, or that an optimal orientation was derived analytically or computationally, regardless of whether PA45° was used as the reference condition; (c) targeted the M1 or the DLPFC; and (d) reported quantitative outcomes (E-field magnitude or component, neuronal activation threshold, MEP amplitude, motor threshold, hemodynamic response, or pain threshold) or a substantive qualitative synthesis of orientation-dependent effects. Records meeting criteria (a) through (c) that provided a qualitative synthesis rather than primary data (narrative or systematic reviews) were not eligible for inclusion as primary studies, but were retained separately as secondary supporting sources. Records were excluded if they were conference abstracts, case reports, editorials, or non-peer-reviewed sources; if coil orientation was held fixed and not examined as a variable; if a non-focal coil geometry was used; or if the cortical target was outside M1 or DLPFC. One exception to the second criterion was made: Yang et al. (2025) held orientation fixed but is retained and reported as contextual evidence on DLPFC network propagation, and is explicitly identified as such wherever it is cited.

Titles and abstracts were screened by two reviewers, and full texts of potentially eligible records were assessed against the criteria above. Data were charted into a standardized extraction form capturing study design, sample size, cortical target, coil and stimulator model, pulse waveform, orientations tested, targeting method, and primary outcome. Disagreements regarding eligibility or classification were resolved by discussion between the authors, while adjudicating where consensus was not reached.

All included articles were organized into two primary categories: computational modeling studies and human experimental studies. Literature reviews retrieved by the searches were not treated as primary evidence and were retained separately as secondary supporting sources. Within these categories, studies were further classified according to the cortical regions investigated, primarily M1 and DLPFC. Within both cortical regions, computational modeling studies were evaluated primarily for predicted E-field distribution and induced-current direction relative to cortical anatomy, derived from MRI-based head models using numerical methods, most commonly the finite element method (FEM) or the boundary element method (BEM); these studies compute field distributions in anatomical models rather than measuring physiological responses in participants (Numssen et al., 2026; Lohr et al., 2026). Human experimental studies were assessed on measured physiological and clinical outcomes: MEP amplitude, resting and active motor thresholds, evoked EEG and hemodynamic responses, and reported therapeutic effects, obtained from participants receiving stimulation. Review articles were not counted among the included studies and were not charted as primary evidence; they were retained separately as secondary supporting sources because they synthesize the primary literature and provide broader interpretation.

For clarity, we define coil orientation throughout this review as the direction of the coil handle relative to the interhemispheric longitudinal fissure, viewed from above the vertex with the anterior direction oriented upward (shown in Figure 1). Angles increase counterclockwise from PA0° for right-hemisphere stimulation and clockwise from PA0° for left-hemisphere stimulation, so that a given PA value denotes the same anatomical relationship in either hemisphere. Reported orientations were re-expressed in this PA convention regardless of reference frame used in the original study, so that eligibility did not depend on whether a study adopted PA45° as its reference condition. All records previously excluded under this criterion were re-screened against the revised criteria. None became eligible, as each remained excludable under at least one other criterion, most commonly that coil orientation was half fixed rather than compared. The included counts are therefore unchanged. Coil-handle orientation and induced-current direction are related but not interchangeable: the direction of current induced in the cortex depends on the coil’s internal winding direction, the polarity of the stimulator output, the stimulator’s configuration, and whether the pulse is monophasic or biphasic (since a biphasic pulse induces current in both directions within a single pulse) with the physiologically dominant phase determined by the waveform. Where studies reported induced-current direction rather than coil-handle position, values were converted assuming a standard biphasic pulse configuration and the manufacturer-reported winding convention of the coil used in each study, and the conversion is noted in the corresponding evidence table; monophasic-pulse studies are noted individually where this assumption does not directly apply. Orientations separated by 180° (e.g., PA45° and PA225°) were treated as distinct conditions rather than equivalent, as they correspond to opposite induced-current directions and, per computational modeling discussed below (Abbasi et al., 2024), can produce different activation thresholds despite sharing the same coil-handle axis.

**Figure 1:**
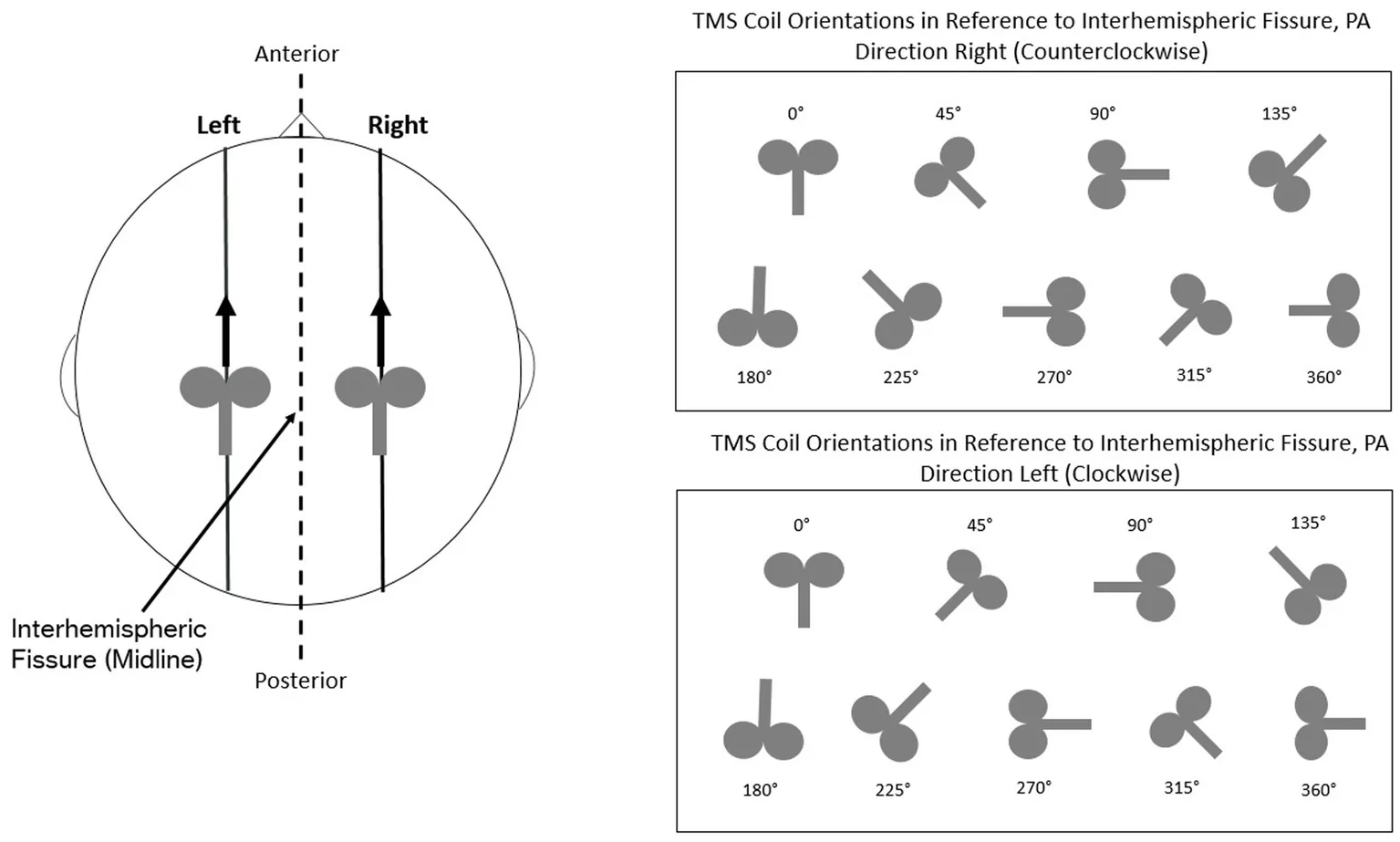
Standardized PA coil orientation convention was used throughout this review, expressed in degrees relative to the PA0° reference position along the interhemispheric fissure. For the right hemisphere, orientation increases counterclockwise from PA0°, whereas for the left hemisphere, it increases clockwise from PA0°, as illustrated by the representative figure-of-eight coil positions shown in 45° increments from PA0° to PA315°. All coil orientations reported in the reviewed literature were converted to this standardized system to enable direct comparison across studies.

Risk of bias in the 18 experimental and physiological studies involving human participants was assessed using ROBINS-I V2 (Sterne et al., 2016), applying its six domains: bias due to confounding, bias in classification of interventions, bias in selection of participants, bias due to missing data, bias in measurement of the outcome, and bias in selection of the reported result. For each study, the target trial was specified as a hypothetical randomized comparison of one coil orientation (intervention strategy) against an alternative orientation (comparator strategy) at the same cortical target, with MEP amplitude, resting motor threshold (RMT), or pain threshold as the outcome. Because the included studies used within-subject designs without post-baseline protocol deviations, Domain 1 Variant A, which addresses baseline confounding only, was applied throughout. Important confounding factors specified in advance were: the order or sequence of orientation testing within a session, time of day of testing, baseline corticospinal excitability at the start of the session, precision of coil placement relative to the individually mapped motor hotspot, participant arousal and attention state, coil-to-cortex distance, and, for DLPFC and pain-related studies, baseline mood or pain state and expectation effects. Given the scope of this review, an abbreviated version of ROBINS-I V2 full signaling-question set was used, applying the single most decision-relevant signaling question per domain rather than the complete battery. Domain-level judgements were assigned as Low, Moderate, Serious, or Critical risk of bias, and the overall judgement for each study was derived using ROBINS-I V2 default algorithm, in which the overall rating equals the rating of the domain judged at greater risk of bias; these are reported in Table 3, with the justification for every domain judgement provided in Supplementary Table S1. ROBINS-I was not applied to computational or E-field modeling studies, as these involve no human participants and no validated risk-of-bias instrument currently exists for this study type, nor to Siebner et al. (2009), Galhardoni et al. (2015), or Sondergaard et al. (2021), which are secondary reviews rather than primary studies. It was likewise not applied to Wu et al. (2025), which, although based on individualized head models derived from participant MRI, reports simulated E-field outcomes rather than measured physiological responses.

**Table 3:** Risk-of-bias assessment of the human experimental and physiological studies included in this review, using ROBINS-I V2.

Study	Target/outcome assessed	D1 Confounding	D2 Classification	D3 Selection	D4 Missing data	D5 Outcome measurement	D6 Selective reporting	Overall
Brasil-Neto et al. (1992)	M1 hand/MEP, RMT	Moderate	Low	Low	Low	Moderate	Moderate	Moderate
Fox et al. (2004)	M1 hand/PET activation	Moderate	Low	Serious	Low	Moderate	Moderate	Serious
Opitz et al. (2013)	M1 hand/MEP	Low*	Low	Low	Low	Moderate	Moderate	Moderate
Richter et al. (2013)	M1 hand/RMT	Low*	Low	Moderate	Low	Low	Moderate	Moderate
Bashir et al. (2013)	M1 hand/MEP	Moderate	Low	Low	Low	Moderate	Moderate	Moderate
Bungert et al. (2017)	M1 hand/MT	Low*	Low	Moderate	Low	Moderate	Moderate	Moderate
Laakso et al. (2018)	M1 hand/MT	Moderate	Low	Low	Low	Moderate	Moderate	Moderate
de Goede et al. (2018)	M1 hand/MEP, TEP, LICI	Moderate	Low	Moderate	Moderate	Moderate	Moderate	Moderate
Souza et al. (2018)	M1 hand/MEP, HD-EMG centroid	Moderate	Low	Low	Low	Moderate	Moderate	Moderate
Hand et al. (2020)	M1 lower limb/RMT, AMT, SICI, SICF	Moderate	Low	Serious	Serious	Moderate	Moderate	Serious
Tervo et al. (2020)	M1 hand/MEP	Low*	Low	Low	Moderate	Moderate	Moderate	Moderate
Weise et al. (2020)	M1 hand/MEP, RMT	Low*	Low	Moderate	Moderate	Moderate	Moderate	Moderate
Gomez-Tames et al. (2021)	Broca’s area, M1/perception and pain thresholds	Moderate	Low	Low	Low	Moderate	Moderate	Moderate
Tani et al. (2021)	Broca’s area, M1/pain threshold, discomfort	Low*	Low	Low	Low	Moderate	Moderate	Moderate
Casula et al. (2022)	Pre-SMA/TEP, LMFP, TRSP	Low*	Low	Low	Low	Moderate	Moderate	Moderate
Becker et al. (2025)	M1 lower limb/MEP	Serious	Low	Low	Low	Serious	Moderate	Serious
Thomson et al. (2013)	DLPFC/HbO (NIRS)	Moderate	Low	Moderate	Low	Moderate	Moderate	Moderate
Yang et al. (2025)	DLPFC/HbO, APA latency and amplitude	Moderate	Low	Moderate	Moderate	Moderate	Moderate	Moderate

The six domains. *D1: Bias due to confounding*. Whether factors present before stimulation began could independently predict both which orientation was applied and the size of the response. In this literature the dominant concern is testing order: if orientations were delivered in a fixed sequence, cumulative stimulation effects or within-session drift in corticospinal excitability cannot be separated from the effect of orientation itself. Pre-specified confounding factors are listed in the Methods. *D2: Bias in classification of interventions*. Whether the coil orientation actually delivered was accurately known and recorded. Studies using neuronavigation, robotic positioning, or a physically calibrated frame were rated low; studies in which the delivered angle could have drifted from the nominal angle without detection were rated higher. *D3: Bias in selection of participants into the study or into the analysis*. Whether participants, sessions, or trials were excluded from the analysis for reasons arising after stimulation began. The principal concern in orientation research is exclusion of participants who could not produce a measurable response, since responsiveness is itself related to the outcome. *D4: Bias due to missing data*. Whether outcome, intervention, or confounder data were incomplete, and whether the missingness could plausibly be related to the true value of the outcome. *D5: Bias in measurement of the outcome*. Whether the outcome was measured in a way that could differ systematically across orientations. Because the operator positioning the coil necessarily knows the orientation, blinding is rarely achievable; studies using fully automated extraction were rated moderate, and studies involving subjective or retrospective judgement of responses were rated higher. *D6: Bias in selection of the reported result*. Whether the reported result was selected from among multiple measurements or analyses. In the absence of a prospectively registered protocol this possibility cannot be excluded, which is why this domain is rated moderate throughout.

The judgement categories (as defined by ROBINS-I V2): *Low risk of bias*: little or no concern about bias in this domain. *Moderate risk of bias*: some concern about bias, although it is not clear that there is an important risk of bias. *Serious risk of bias*: the study has important problems in this domain that give rise to a serious risk of bias. *Critical risk of bias*: the study is so problematic in this domain that the result should generally be excluded from evidence syntheses. No included study received this rating. *Overall judgement*. Derived using the ROBINS-I V2 default algorithm: the overall rating for a study equals the rating of the domain judged at greatest risk of bias. No override of the algorithm was applied.

*In Domain 1, ROBINS-I V2 designates the lowest available rating as “low risk of bias except for concerns about uncontrolled confounding,” reflecting that residual confounding can never be fully excluded in a non-randomized design. Cells marked with an asterisk carry this meaning. Domain-level justifications for every judgement are provided in Supplementary Table S1. ROBINS-I was not applied to the computational and E-field modeling studies, as these involve no human participants and no validated instrument currently exists for this study type, nor to the secondary reviews reported in Table 7.

AMT, active motor threshold; APA, anticipatory postural adjustment; DLPFC, dorsolateral prefrontal cortex; HbO, oxygenated haemoglobin concentration; HD-EMG, high-density electromyography; LICI, long-interval intracortical inhibition; LMFP, local mean field power; M1, primary motor cortex; MEP, motor evoked potential; MT, motor threshold; NIRS, near-infrared spectroscopy; PET, positron emission tomography; pre-SMA, pre-supplementary motor area; RMT, resting motor threshold; ROBINS-I, Risk Of Bias In Non-randomized Studies – of Interventions; SICF, short-interval intracortical facilitation; SICI, short-interval intracortical inhibition; TEP, TMS-evoked potential; TRSP, TMS-related spectral perturbation.

## Results

A total of 1,200 records were initially identified for M1-related studies. After removing 393 duplicates, 807 articles remained for title and abstract screening. From this stage, 615 articles were excluded because they were not related to coil orientation, did not address coil orientation or positioning parameters, or were related to irrelevant conditions. The remaining 192 full-text articles were retrieved for detailed eligibility assessment. Of these, 168 were excluded upon full-text review for one or more of the following reasons: they did not specify coil orientation relative to M1 or DLPFC; coil orientation was held fixed rather than evaluated as an independent variable; a non-focal coil geometry was used; or they did not provide mechanistic, computational, or experimental conclusions regarding orientation-dependent stimulation effects. This process yielded 24 final studies for the motor cortex: 8 computational modeling studies, 16 experimental studies. The majority of experimental studies focused on motor physiology rather than pain outcomes; only one article directly examined coil orientation in relation to pain modulation (Figure 2).

**Figure 2:**
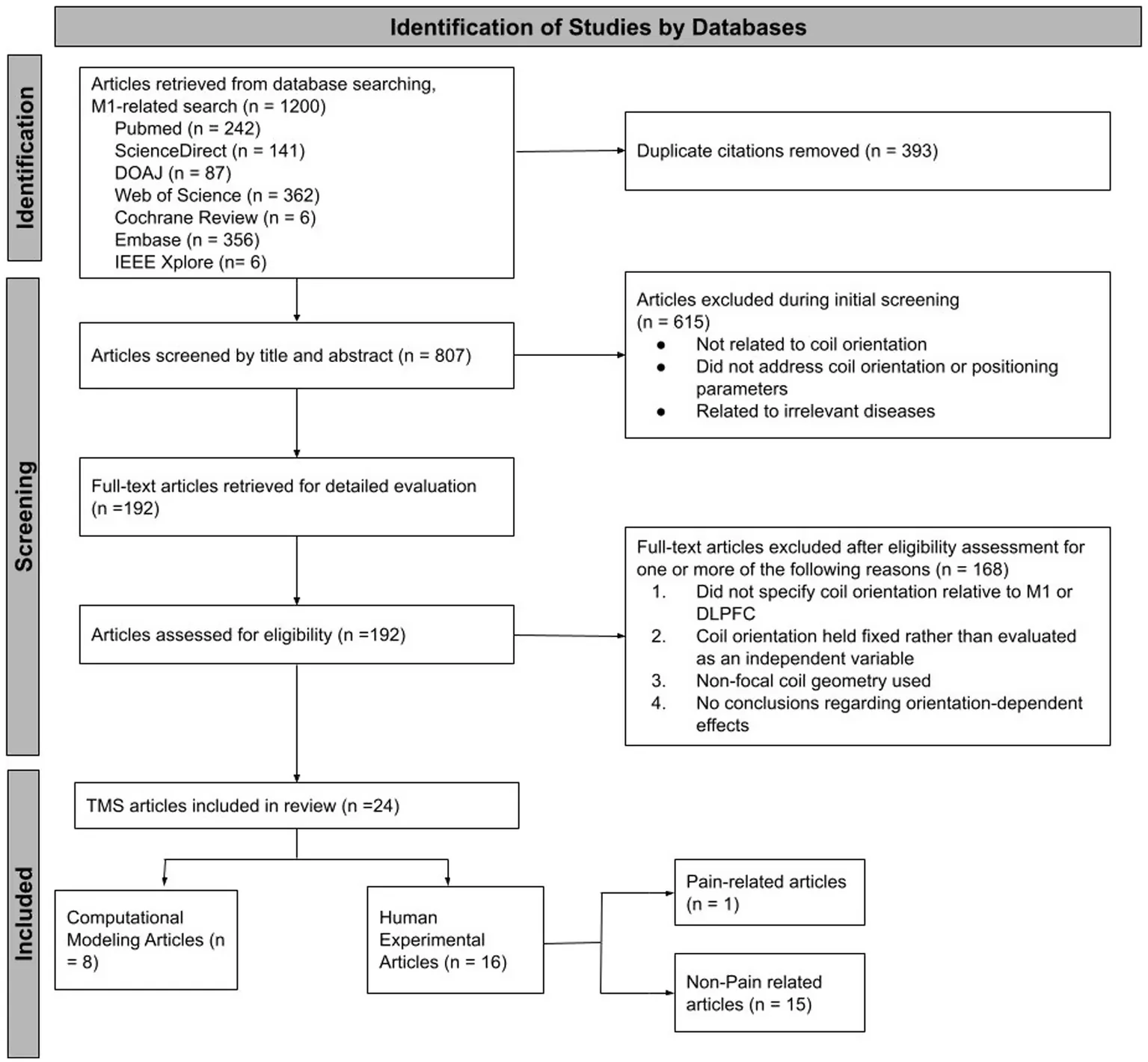
PRISMA flow diagram for the M1-related literature search. DOAJ, Directory of Open Access Journals; M1, Primary Motor Cortex; TMS, Transcranial Magnetic Stimulation.

For the DLPFC-related, 86 articles were initially identified across the databases. Fifty-three were removed as duplicates, either within the DLPFC search (*n* = 22) or because they had already been retrieved by the M1 search (*n* = 31), leaving 33 records for full-text assessment. Twenty-seven were excluded because they did not address orientation at the DLPFC or did not provide conclusions regarding coil orientation, and a further three were excluded because they did not report an outcome attributable to orientation, giving 30 exclusions in total. Three records met the eligibility criteria: one mixed computational and experimental E-field modeling study (Wu et al., 2025) and two experimental studies (Thomson et al., 2013; Yang et al., 2025). Of the two full experimental studies, only Thomson et al. manipulated coil orientation; Yang et al. delivered stimulation at a single fixed mediolateral orientation and is included as contextual evidence on network-level propagation rather than as an orientation-manipulation study (Figure 3).

**Figure 3:**
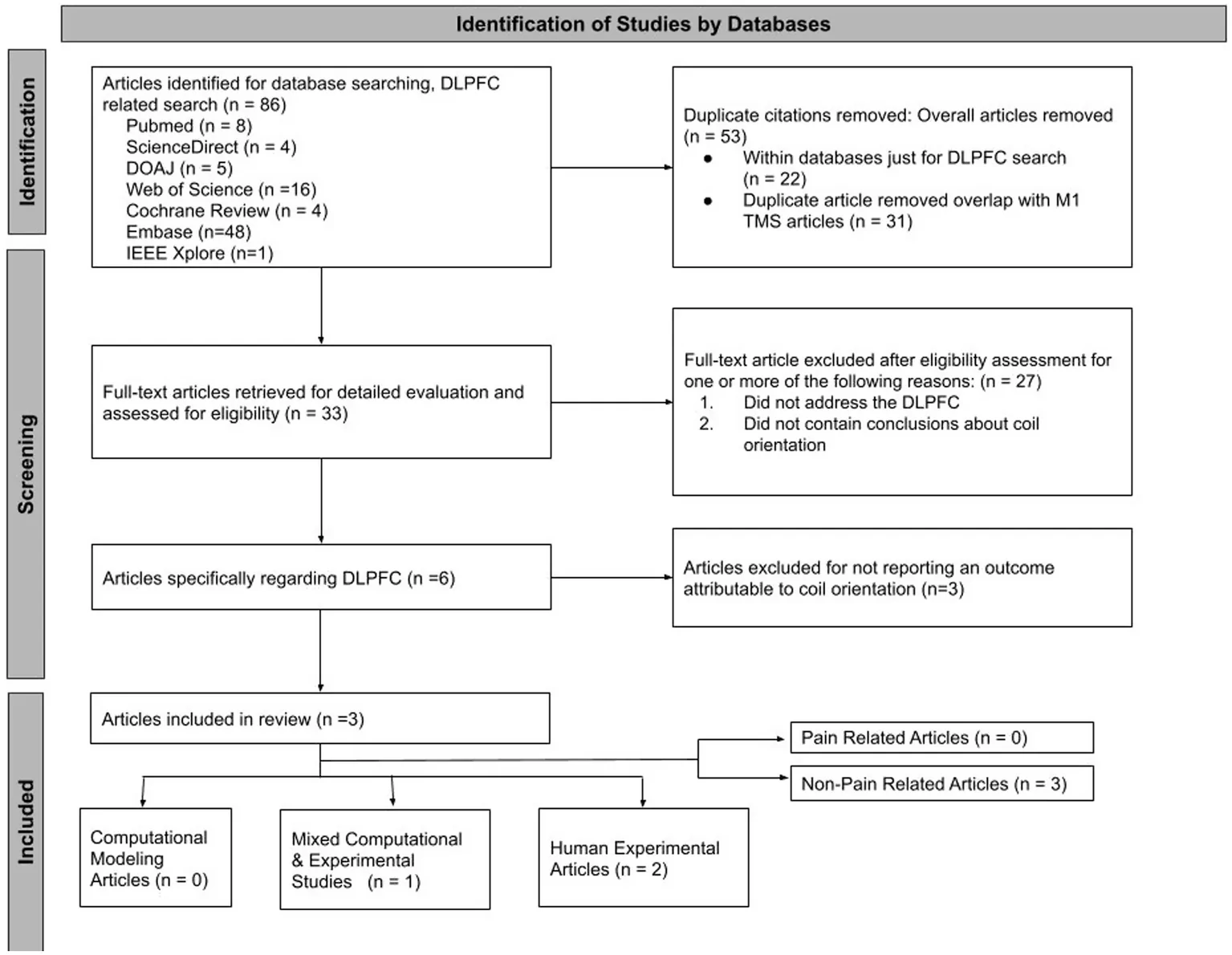
PRISMA flow diagram for the DLPFC-related literature search. DOAJ: Directory of Open Access Journals; M1, Primary Motor Cortex; TMS, Transcranial Magnetic Stimulation; DLPFC, Dorsolateral Prefrontal Cortex.

### Risk of bias assessment: ROBINS-I V2

Risk-of-bias judgements for the 18 experimental/physiological studies are summarized in Table 3. The most frequently flagged domain was measurement of the outcome, reflecting the general absence of blinded MEP assessment across this literature, a structural feature of the field rather than a flaw specific to individual studies. Confounding was rated Low or Moderate across the majority of studies. Studies that explicitly randomized or counterbalanced the order in which orientations were tested were rated low in this domain. Where testing order was fixed, or where it was not reported, a Moderate rating was assigned, leaving open possibility that cumulative stimulation effects or within-session drift in corticospinal excitability contributed to apparent orientation effects.

### Optimal coil orientation based on computational modeling at M1

Overall, in the 8 selected articles assessing E-field modeling at M1, the majority of them suggest that the strongest E-fields and lowest motor threshold could be achieved when the induced field was oriented perpendicular to the local cortical anatomy of the central sulcus. For M1, the optimal angle generally fell between approximately PA45° and PA70°, whereas cortical regions outside the M1 area, including the pre-SMA, required substantially different orientations (Table 4).

**Table 4:** Computational modeling studies examining coil orientation effects in the primary motor cortex (M1).

Author and Year	Type of coil used	Aim	Methods	Results	Conclusion
Nummenmaa et al. (2014)	FoE	Optimizes TMS stimulation of a specific axonal bundle in the motor cortex.	BEM, Brain MRI and FOE coil rotated PA 0–170° (10° steps), over lateral hand M1.	PA90° produced the strongest E-field gradients, while PA ~ 70° generated the most focal maximal stimulation, with activation declining rapidly when the coil deviated from this angle.	TMS activation strongly depends on precise coil orientation and positioning, with a focal optimal angle (~70°) for a specific axonal bundle.
Janssen et al. (2015)	FoE	Determines the optimal coil orientation for the inward perpendicular E⊥.	FEM, C^3^ model and FOE coil at 14 cortical targets at 36 coil angles (10° steps) at M1.	PA45° coil orientation maximized E⊥ and produced the most stable MEPs, whereas rotating toward PA90° reduced E⊥ and response amplitude.	PA45° orientation optimally aligned E⊥ with C^3^ model, producing stronger, more reliable MEPs.
Gomez-Tames et al. (2018)	FoE	Identify anatomy-dependent optimal coil position/orientation across cortex using large-scale modeling.	18 MRI-based head models observed 14 segmented tissue types. E-fields computed using magneto-quasi-static volume conductor model across 518,616 simulation scenarios (2,401 scalp locations ×12 orientations at 15° intervals).	Maximum E-fields were strongest in M1 and S1, with optimal coil orientation aligning perpendicular to local gyrus anatomy (R^2^ = 0.91); field strength decreased sharply with scalp–cortex distance, and optimal scalp positions varied slightly (~5.5 mm) from the closest cortical point.	Optimal coil orientation is anatomy-dependent rather than a fixed PA45°, especially outside sensorimotor cortex, and anatomy-guided atlases can improve personalized TMS targeting.
Lee et al. (2018)	FoE	Assess how CSF thickness, BSD, and coil orientation influence inter- and intra-subject variability at the vertex.	FOE coil over vertex on 50 subject-specific models: PA0°, PA90°, PA 180° rotations; using E-max & V-half as focality metrics	Greater BSD and thicker CSF reduced peak electric-field strength and increased field spread. Optimal coil orientation varied by target, with M1 strongest at ~PA90° and SMA strongest at PA0°/180°.	Both anatomical factors (CSF and BSD) and coil angle independently shape TMS F-field intensity and focality.
Gomez et al. (2021)	FoE	Develop fast ADM to optimize coil orientation maximizing E-field & quantify placement uncertainty.	FEM, ADM, Brain MRI-driven ROI and FOE coil in 2.1 million coil configurations (5,900 scalp positions × 360 orientations) using a fast-multipole method and a FOE coil model approximated by fewer than 600 auxiliary dipoles.	ADM optimization shifted coil placement 1–14 mm from conventional neuronavigation targets and increased the ROI electric-field magnitude by up to 6%. Position errors strongly reduced field strength, while orientation errors produced smaller effects.	Accurate position is more critical than exact angle; systematic exploration beyond standard PA-45° enables subject-specific optimization.
Makarov et al. (2021)	FoE	Determine optimal E-field focality at M1	BEM, FMM, and TMS-IP solutions to evaluate the degree of improvement of the TMS-IP solutions relative to the well-known sulcus-aligned mapping	The TMS-IP solutions with the maximized focality simultaneously improve the TMS mapping resolution	The accuracy of the head segmentation critically influences the expected TMS-IP performance.
Abbasi et al. (2024)	FoE	Determine how coil orientation and pulse parameters affect neuronal activation thresholds	Multiscale computational simulation of a layer-5 pyramidal neuron placed in the left M1 hand knob within a realistic head model, using a FOE coil at 24 orientations PA (0–345°, 15° steps)	Modeling showed lowest neuronal activation thresholds at coil orientations P45°/P225°, where the induced electric field aligned with the pyramidal axon. Orientations around P90°/P270° produced the highest thresholds.	Coil orientation and pulse parameters could jointly determine stimulation efficiency orientation. Shorter pulse widths increased thresholds but did not change the optimal orientation.
Laakso et al. (2025)	FoE	Quantify differences in cortical activation sites between PA and AP TMS.	Probabilistic and brain MRI models were correlated with MEPs from 5 hand/arm muscles in 9 healthy participants using monophasic FOE PA and AP stimulation.	Both PA and AP TMS preferentially activated white matter, but PA sites localized mainly to the precentral gyrus (white/gray matter interface), whereas AP sites were deeper and more posterior/lateral; reversing current direction shifted activation several millimeters.	Current direction determines which neural elements are recruited, with PA and AP TMS engaging spatially distinct cortical regions and shifting the likely activation site by several millimeters.

TMS, Transcranial Magnetic Stimulation; BEM, Boundary Element Method; MRI, Magnetic Resonance Imaging; FOE, Figure of Eight coil; M1, Primary Motor Cortex; PA, Posterior-Anterior; E-field, Electric Field; E⊥, E-Field Component; FEM, Finite-Element Modeling; C^3^ Model, Cortical Column Cosine Model; MEP, Motor Evoked Potential; S1, Primary Somatosensory Cortex; CSF, Cerebrospinal Fluid; BSD, Brain-Scalp Distance; E-max, Maximum Electric Field; V-half, Half-Max Stimulated Volume; SMA, Supplementary Motor Area; ADM, Auxiliary Dipole Method; ROI, Region of Interest; FMM, Fast Multipole Method; P, Posterior; TMS-IP, Transcranial Magnetic Stimulation Inverse Problem.

Using a highly realistic head model and FEM, Janssen et al. (2015) calculated the E-field with 14 cortical target locations and 36 coil orientations tested in 10° steps, and demonstrated that when the coil was placed at approximately a PA45° orientation at M1, the induced PA E-field was maximized along its component perpendicular to the anterior wall of the central sulcus, where most corticospinal neurons originated. The cortical column cosine (C^3^) model further supports this by showing that the induced E-field is strongest when it aligns with the direction of cortical columns and becomes weaker as the coil rotates away from this alignment. While small orientation changes of ≤10° did not dramatically alter the induced E-field, larger deviations (>10° from the optimum) caused meaningful reductions in field strength. The same principle appears to be applicable for other cortical areas beyond M1 (Janssen et al., 2015).

The outcomes of the E-field modeling raise the question of how individual cortical anatomical variations may affect the projected E-field.

One of the largest computational studies was performed by Gomez-Tames et al. (2018), whose atlas was constructed from 518, 616 simulated stimulation scenarios, using 18 individualized head models, 2,401 coil positions, and 12 orientations at 15° intervals. The authors found that the optimal coil orientation consistently aligned perpendicular to local gyral anatomy, especially in the motor hand area (*R*^2^ = 0.91). However, the analysis suggested that the maximum E-field was strongest at the gyral crown rather than the sulcal wall, contradicting earlier modeling studies. The strongest and most reliable fields were found in the primary motor cortex, especially the hand area, while occipital regions showed weaker and less consistent E-field projections. This indicates that computation-based E-field projections are most achievable at the primary motor cortex and become progressively less reliable elsewhere. This study also found that optimal coil orientation did not necessarily correspond to the direct coil placement over the cortical target. In the cases of M1 and premotor area, a shift of 5 mm was noted with the overall mean position shift in the cortical surface at 5.5 ± 1.6 mm and larger cortex-scalp distances correlated with larger coil shifts (*R*^2^ = 0.4564, *p* < 0.001) (Gomez-Tames et al., 2018).

Lee et al. (2018) further demonstrated that individual anatomical variations could alter projected E-field by conducting simulations on 50 individualized head models to study inter-subject variability, and 25 models to examine the effects of orientation change intra-subject. Using FEM TMS simulation modeling, the authors found that greater brain-scalp distance (BSD) and thicker cerebrospinal fluid (CSF) were associated with weaker induced stimulation intensity and a more diffuse electric-field spread, with CSF remaining a significant predictor even after accounting for BSD. In their interpretation, thinner CSF may amplify local E-field peaks and thicker CSF may reduce focality, whereas BSD primarily reflected the coil-to-cortex distance that shaped stimulation intensity through magnetic-field decay. Together, these two anatomical parameters accounted for 80% of the variability in peak E-field magnitude. Across subjects, stimulation intensities ranged from 83 to 188 V/m, and field spread ranged from 6.8 to 24.4 cm^3^, showing a nearly threefold difference in both measures, even when the same protocols were used. Coil orientation further contributed to intensity, orienting the coil perpendicular to the longitudinal fissure produced approximately 10% stronger stimulation intensity compared to a parallel orientation (Lee et al., 2018). These findings do appear to support the overall assertion that individual brain anatomical variations can affect the projected E-field.

Expanding to the cortical gray matter level, and using probabilistic and biophysical axon modeling combined with motor threshold data from nine participants across 11 scalp positions with individually derived E-field models, Laakso et al. (2025) demonstrated that TMS induced E-field in both PA and AP directions perpendicular to the precentral gyrus were more likely to activate white matter structure than gray matter. TMS induced PA E-field mainly activated regions in the precentral gyrus near the white–gray matter interface, while AP E-field activated deeper and more posterior white matter regions of the precentral gyri (Laakso et al., 2025). In addition, the study also showed that the optimal coil orientation for eliciting motor responses from different hand muscles, including FDI, APB, extensor carpi ulnaris (ECU), and flexor carpi ulnaris (FCU), averaged approximately 45° to the fissura longitudinalis (PA45°), supporting the conventional rule. Combined with RMT observations, this modeling suggests that PA and AP E-fields can affect the cortical neuronal substrate differently: PA45° remained optimal for eliciting motor responses from most hand muscles relevant to M1 due to the broader but uniform E-field coverage, whereas larger coil orientation angle produced more focal activation (Laakso et al., 2025).

In addition, using diffusion-MRI tractography with Boundary Element Method (BEM) which calculated the E-field generated in the cortex by different coil positions, Nummenmaa et al. (2014) showed that at PA0°, the strongest E-field gradient shifted medially from the point directly beneath the coil, whereas at PA45°, the projected E-field at the M1 covered a relatively large and uniform area in the posterior bank of the precentral gyrus; and at PA90°, E-field gradients were stronger overall, but spatially distinct with the most focal tract-specific activation occurred at approximately PA70° rather than PA45°, suggesting that the optimal angle for activating specific white matter pathways may differ from the conventional default.

#### Computational optimization and targeting precision

Several algorithmic methods have been developed to bridge the gap between the standard fixed 45° approach and the true optimal angle for each individual. For example, Makarov et al. (2021) applied the TMS inverse problem (TMS-IP) framework to analyze 1,536 individual solutions across multiple subjects and target points using the BEM fast multipole method. Their goal was to maximize E-field focus at the specified M1 hand area targets, while minimizing spread to surrounding regions (Makarov et al., 2021). Compared to the standard sulcus-aligned mapping method, which produced an average angle of about 29° toward the fissura longitudinalis cerebri, TMS-IP approach shifted the optimal angle to approximately PA35–36°, bringing it closer to the experimentally supported PA45° motor threshold minimum, while also improving E-field focality by approximately 21–33% on average. Importantly, the study found that the accuracy of head segmentation was the primary determinant of TMS-IP performance, implying that once individual anatomy was accurately characterized, further improvements in precision depended more on the quality of the anatomical model than on making the algorithm itself more complex (Makarov et al., 2021).

At the biophysical scale, Abbasi et al. (2024) developed a multiscale model combining an anatomically realistic macroscale brain cortex model with a layer-5 pyramidal neuron placed in the left M1 hand knob region. The neuron’s axon was oriented perpendicular to the central sulcus to represent cortical column organization (Abbasi et al., 2024). Across all pulse widths, the lowest stimulation thresholds occurred at PA45° and PA225°, where the induced E-field aligned parallel to the neuronal axon, whereas maximum thresholds occurred at PA90° and PA270°, where the E-field was perpendicular to the axon, regardless of pulse width. These results confirm that optimal TMS orientation may be governed by two interacting factors: the three-dimensional geometry of sulci and gyri beneath the coil, and the orientation of cortical columns at the target site, both of which vary with coil location, making universal fixed-angle protocols inherently suboptimal for non-M1 targets (Abbasi et al., 2024) (Figure 4).

**Figure 4:**
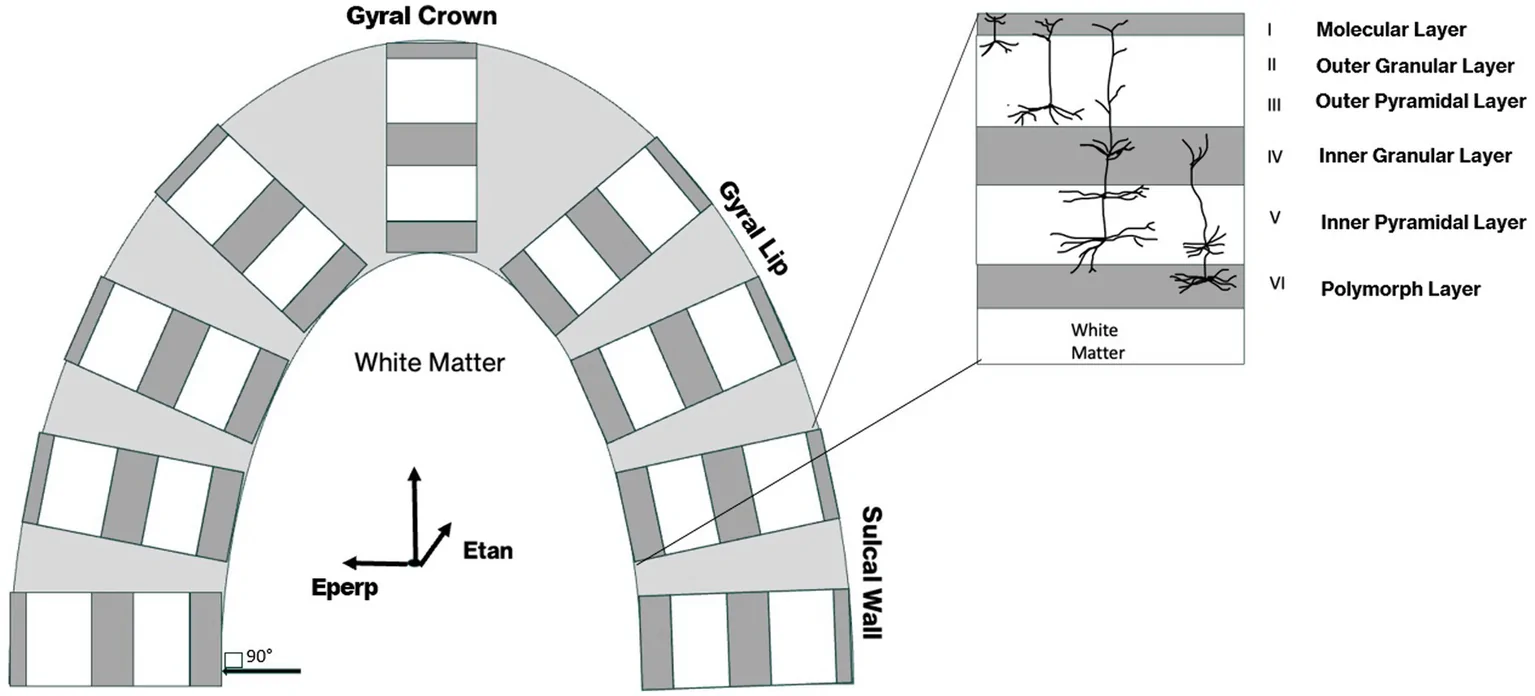
Schematic of cortical column geometry relevant to TMS-induced electric-field orientation, shows columns extended perpendicular to the cortical surface across the gyral crown, gyral lip, and sulcal wall, with cell bodies distributed across cortical layers I–VI above the white matter boundary. TMS is most effective when the induced electric field aligns with local cortical column orientation, which varies across gyral and sulcal regions. The induced electric field vector (E⊥) is shown at 90° to the anterior wall of the central sulcus (the posterior bank of the precentral gyrus) and therefore parallel to the principal axis of the underlying columns and to the pyramidal axon axis at that location. Activation thresholds are lowest in this configuration and highest where the field runs perpendicular to the axon, which is the physical basis for the anatomy-dependent optimal orientation described throughout this review.

### Optimal coil orientation from human experimental studies at M1 and DLPFC in non-pain studies

Several important observations from human experimental assessments, such as motor evoked potential (MEP), further illustrate how physiological outcomes correlated with E-field modeling and known individual anatomical and physiological variables may affect the stimulation outcomes with different coil orientations.

#### Empirical foundation of the PA45° convention

MEP readings, recorded via electromyography (EMG) associated with a brief muscle twitching following each TMS pulse, serve as the primary physiological readout of corticospinal excitability and allow precise quantification of how effectively a given coil angle activates the targeted neurons. Across the experimental studies that targeted the M1 hand representation and used MEP amplitude or motor threshold as the outcome, optimal orientations clustered near PA45°, with reported group means ranging from approximately PA15° to PA50° depending on which intrinsic hand muscle was recorded. This convergence across subjects, hemispheres, and recording methods is the empirical basis of the PA45° convention. It should be read as a statement about the hand representation specifically: as detailed below, studies targeting lower-limb motor cortex, the pre-supplementary motor area, and non-motor regions report optima that depart substantially from this value.

Two early mapping studies support this convergence. Brasil-Neto et al. (1992) systematically tested eight coil orientations spaced 45° apart over the right M1 hand area and found maximal MEPs at PA225° ± 45° and PA30° ± 52° with the optimal induced current direction for activating the left APB representation running approximately perpendicular to the presumed position of the central sulcus and flowed anteriorly. Similarly, Mills et al. (1992) reported peak MEP amplitude at the first dorsal interosseous (FDI) at both left and right M1 was at approximately PA50° (±19)° coil orientation, with smaller responses at all other orientations. Combining electrophysiological recordings with FEM, Bungert et al. (2017) and Laakso et al. (2018) subsequently confirmed that the PA45° configuration produced both the lowest motor thresholds and the strongest E-field at the gyral crown of the M1 hand-knob region. PET imaging provided independent confirmation, localizing TMS-induced cortical activation selectively on the sulcal bank of the central sulcus at approximately 31 mm depth, matching voluntary movement areas and consistent with C^3^ model predictions (Fox et al., 2004). The convergence of these experimental, computational, and imaging findings confirms that the PA45° orientation is not an arbitrary convention but an approximation of the angle that best aligns the induced E-field with the predominant axis of pyramidal neurons in the anterior sulcal wall.

#### Orientation-dependent motor responses across muscles and hemispheres

In assessing how coil orientation may affect hand muscle motor response, Souza et al. (2018) tested eight orientations spaced 45° apart from PA0° to PA315° using a high-density 61-electrode grid over the right APB muscle with left M1 stimulation and found that MEP amplitudes were largest at PA45° and PA90°, while the smallest amplitudes were elicited at PA135° and PA315°, in orientations parallel to the central sulcus. Critically, the spatial center of MEP activity over the muscle stayed the same across orientations, potentially showing that coil orientation mainly changed top-down corticospinal excitability strength but did not affect the cortical region.

Using MRI-based neuronavigation with fine angular resolution, Bashir et al. (2013) mapped optimal current directions for three intrinsic hand muscles: FDI, APB, and abductor digiti minimi (ADM), in ten healthy subjects, rotating the coil in 45° increments (PA oriented) and then refining to 5° steps around the identified optimum. Mean optimal angles for the left hemisphere were PA35.5° ± 6.75° (FDI), PA15.6° ± 11.68° (APB), and PA32.4° ± 15.0° (ADM), and right hemisphere values were comparable (PA30.1°, PA9.0°, and PA32.2° for FDI, APB, and ADM respectively). In four subjects retested on a separate occasion, optimal directions remained highly consistent, with inter-session variability as low as 2.0° for FDI and 0.5° for APB (Bashir et al., 2013). These results demonstrate that while 45° captures the central tendency of optimal orientations, each muscle has a subtly distinct preferred direction reflective of its unique pyramidal and intraneuronal organization, and that MRI-guided neuronavigation can help resolve these differences.

#### Individual-level sensitivity to orientation and position

At the individual level, sensitivity to orientation changes is substantial and largely masked by group-level averaging. de Goede et al. (2018) used a robot-guided navigation system to test eight scalp positions at 2 mm and 5 mm distances around the initially identified hotspot and evaluated the effect of ±10° coil rotation on MEP amplitude, TMS-evoked potentials (TEPs), and long-interval intracortical inhibition (LICI). In all eight subjects, the highest MEP was not at the hotspot originally identified by standard function-guided navigation, confirming that conventional methods may systematically underestimate the true optimum. Group averages showed no significant effects of position or orientation on MEP, TEP, or LICI, however, at the individual level, significant effects were present for both changes in position and orientation (de Goede et al., 2018). The outcomes of these studies suggest subject-specific variability in MEP does exist.

Furthermore, Opitz et al. (2013) recorded MEPs at 25 discrete scalp locations using two coil orientations, PA45° and PA90°, in five healthy subjects, and compared responses against individually computed E-field simulations. While no significant group-level difference in mean MEP amplitude between the two orientations (PA45°: 897.80 ± 257.70 μV; PA90°: 805.40 ± 435.31 μV; *p* = 0.41) was observed, within-subject comparisons revealed consistent individual preferences. For instance, two subjects showed significantly larger MEPs at PA90°, while three showed significantly larger MEPs at PA45°. FEM E-field strength correlated strongly with empirically measured MEP amplitudes, and the optimal orientation for each subject depended on hand-knob morphology of an inverted-omega shape, which favored PA45°, whereas an epsilon shape favored PA90° (Figure 5). Coil rotation was the dominant driver of E-field distribution shifts within that dataset, indicating that orientation is an influential and directly controllable parameter in stimulation targeting (Opitz et al., 2013). Across the broader literature, inter-individual variability in optimal orientation at M1 alone can span as much as ±30° (Lee et al., 2018; Goodwin and Butson, 2015).

**Figure 5:**
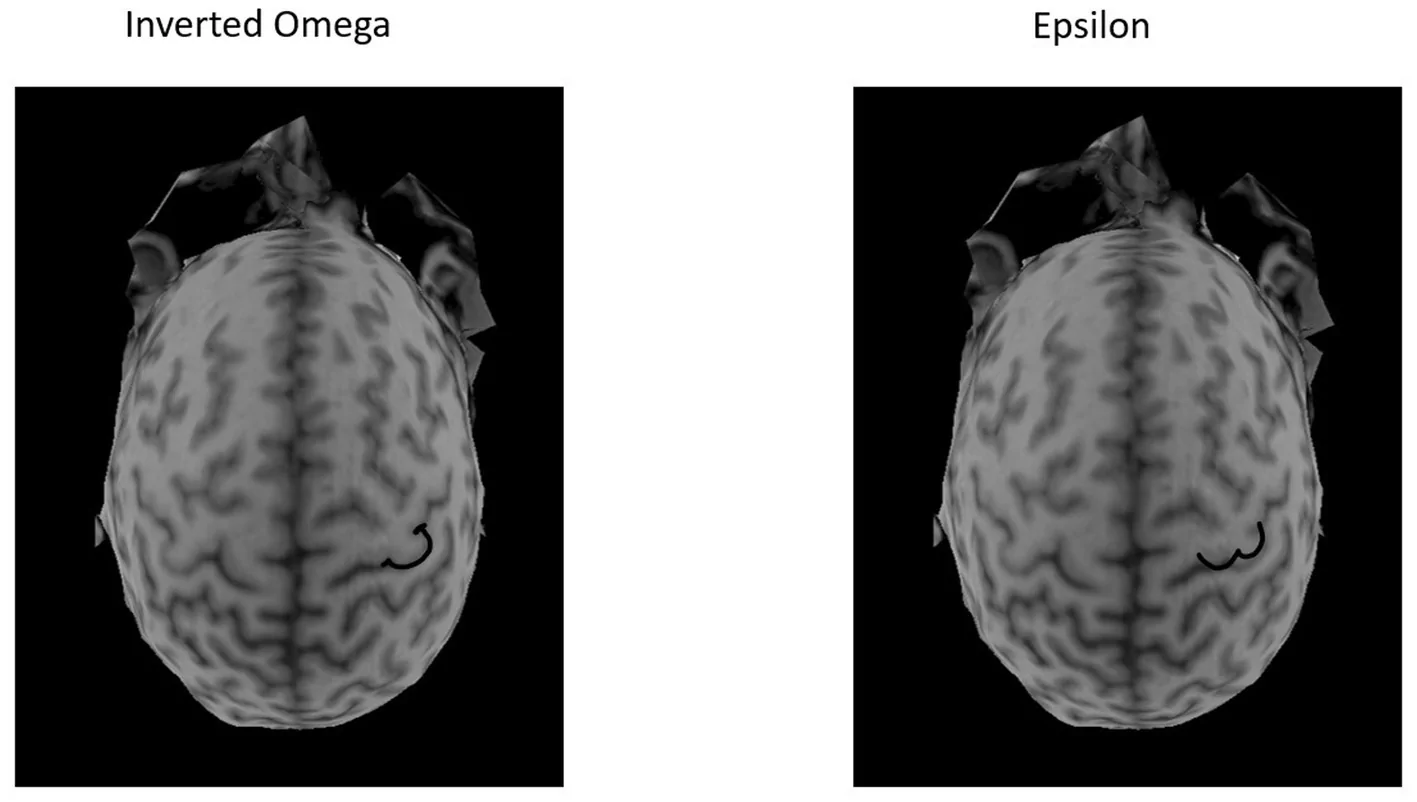
Two common morphologies of the precentral gyrus “hand knob”: an inverted-omega shape and an epsilon shape. This landmark identifies the hand representation of the primary motor cortex, and its morphology is associated with the coil orientation producing the largest motor evoked potential, with the inverted-omega configuration favouring approximately PA45° and the epsilon configuration favouring approximately PA90°. Identifying hand-knob morphology on an individual MRI can therefore help guide coil placement and orientation for TMS. Original schematic prepared by the authors, adapted from simulated T1-weighted MRI volumes obtained from the BrainWeb Simulated Brain Database, McConnell Brain Imaging Centre, Montreal Neurological Institute, McGill University.

#### Orientation effects beyond the hand representation

The anatomy-dependent nature of optimal coil orientation becomes most evident when stimulation targets shift away from the M1 hand knob, where cortical geometry diverges substantially from the central sulcus template that underlies the PA45° convention.

Richter et al. (2013) demonstrated that the mean RMT difference between the optimal and two conventional coil orientations was 8.0 ± 5.9% and 11.8 ± 10.8%, respectively, while the optimal coil angles averaged PA33.1° ± 18.3° and PA213.3° ± 12.1°, respectively. These results indicate a potential loss in clinical efficacy when the standard angle is used without individual adjustment. On the other hand, optimal orientations correlated significantly with the direction perpendicular to the precentral gyrus at the hot spot, supporting the idea that coil orientation should follow cortical anatomy rather than a fixed scalp-based rule; this differs from the hand motor cortex, where the knobby geometry and variable column directions make orientation less uniform (Richter et al., 2013).

For the tibialis anterior (TA) muscle located at the front compartment of the lower leg, the deviation from M1 hand conventions is even more pronounced. The study by Hand et al. (2020) compared PA0° and PA270° during both rest and voluntary contraction and found that with left hemisphere stimulation, the resting motor threshold (RMT) for TA was significantly lower at PA270° in comparison to PA0° stimulation (*p* < 0.05). Beyond the difference in RMT, the two orientations produced qualitatively different intracortical excitability profiles: at rest, the PA270° oriented coil produced greater short-interval intracortical inhibition (SICI) and less short-interval intracortical facilitation (SICF) compared with the PA0° oriented coil. During muscle activation, this pattern reversed, with the PA270° oriented coil producing reduced SICI and increased SICF (Hand et al., 2020). This orientation × activation state interaction has no equivalent in hand M1 studies, indicating that lower-limb motor cortex stimulation physiology cannot be inferred from hand-area observations. Such findings were further extended by Becker et al. (2025), using neuronavigation-guided mapping with eight stimulation directions at 45° increments across six scalp positions at the left M1 in PA configuration, finding that the TA motor hotspot was located in the precentral gyrus 6–13 mm lateral to the midline. Additionally, peak MEP amplitudes occurred at PA90°, perpendicular to the interhemispheric plane (falx cerebri), with PA90° and PA135° producing significantly higher amplitudes than orientations between PA225° and PA315° (Becker et al., 2025). The amplitude distribution followed a sinusoidal pattern with a clear peak at PA90°, confirming that the optimal orientation for the lower limb at the precentral gyrus is systematically different from the PA45° convention and predictable from the underlying cortical anatomy.

The principle that optimal orientation follows local cortical geometry extends to white matter pathway targeting. As described in the computational section, Nummenmaa et al. (2014) demonstrated that the most focal tract-specific activation within left M1 occurred at approximately at PA70° rather than PA45° (left PA coil configuration), suggesting that generalizing the hand-area PA45° convention to other motor targets may result in systematic and reducible error. A well-characterized example of how far optimal coil orientation can depart from M1 norms is the pre-supplementary motor area (pre-SMA). Casula et al. (2022) applied TMS at multiple orientations over the right pre-SMA in combination with simultaneous EEG recording and found that at PA0° orientation produced the most robust and selective pre-SMA activation in TMS-evoked EEG responses, while at PA90° coil orientation increased overall stimulation potency at the cost of spatial focality, spreading activation into adjacent M1, suggesting stimulation potency and spatial resolution did not necessarily correlate with each other (Table 5).

**Table 5:** Experimental studies examining coil orientation effects in the motor cortex.

Author and year	Type of coil used	Aim	Subject (N)	Methods	Results	Conclusion
Brasil-Neto et al. (1992)	FoE	Assess how coil orientation, intensity, and pulse shape affect MEP amplitude	10	MEPs were obtained at coil orientations PA45°–315°, using a FOE coil at RMT + 10–20% or maximal stimulator output	Lowest RMT and largest MEP occurred when induced current flowed diagonally posterolateral-to-anteromedial (~45° relative to the midsagittal plane), approximately perpendicular to the central sulcus.	A ~ 45° PA-directed current maximizes corticospinal activation and defines the classic orientation rule.
Fox et al. (2004)	FoE	Determine TMS-induced specific cortical locational activations	12	PET for detecting locational cortical activities with C3 E-field modeling and B-shaped butterfly coil providing real and sham stimulations with or without voluntary movement.	TMS-induced cortical activation occurred selectively on the sulcal bank of the central sulcus (~31 mm depth), matching voluntary movement areas.	~PA 45° coil orientation, E-field best aligns perpendicularly E-field with the sulcal wall; optimal excitation occurs when the induced field is longitudinal to neural elements and less when transverse.
Opitz et al. (2013)	FoE	Assess whether FEM better predicts the optimal coil orientation than conventional approaches	5	Subjects received single-pulse biphasic TMS at 120% resting motor threshold (MT) using a 35 mm FoE coil positioned at PA45° and PA90° relative to the midline.	FEM-predicted strong E-field and MEP amplitude correlations. Individuals with inverted-omega hand-knob showed larger MEPs at PA45°; participants with an epsilon-shaped hand-knob responded better at PA90°.	FEM can better predict optimal E-Field projections with morphological hand-knob variation than conventional approaches.
Richter et al. (2013)	FoE	Compared 2 conventional orientations (PA90°, PA 270°) with optimal coil orientation of resting MT measurements at right M1.	8	RMTs were determined at the various coil orientations near the two conventional orientations.	The mean RMT difference between the optimal and two conventional coil orientations was 8.0 ± 5.9% and 11.8 ± 10.8% respectively, while the optimal coil angles averaged PA33.1° ± 18.3°and PA213.3° ± 12.1°, respectively.	This result indicates that a potential loss in clinical efficacy when the standard angle is used without individual adjustment for gyral morphology.
Bashir et al. (2013)	FoE	Identify the optimal TMS coil orientation for three intrinsic hand muscles (FDI, APB, ADM)	10	FoE coil delivered at 110% of individual RMT from PA 45° to 315° around the hotspot. For each orientation 10 MEPs were recorded per muscle.	Optimal angles for FDI, APB, and ADM: In the left hemisphere, FDI and APB responses peaked near approximately 45° to central sulcus while ADM peaked near 0°. In the right hemisphere all three muscles were optimally activated near PA45.	Optimal coil orientation for MEPs can be hemispheric and muscle group specific, as shown by variation in muscles activated between the two hemispheres. Such results were claimed to be reproducible.
Bungert et al. (2017)	FoE	Identify which M1 subregion is stimulated via TMS by combining electrophysiological thresholds with realistic FEM E-field estimates.	9	9 coil orientations (−90° to +90°, 22.5° increments PA45 orientation); individualized FEM head models were used to extract both total E-field magnitude and its normal component in gray matter, white matter, and CSF.	PA45° orientation produced the lowest motor thresholds and the strongest E-field at the gyral crown of the M1-hand knob region. PA45 coil orientation optimally aligns the induced E-field with hand-knob cortical geometry,	PA45 coil orientation changes can maximize field strength at gyral crown and minimize motor threshold, suggesting coil orientation relative to individual cortical anatomy as a critical determinant of stimulation efficiency.
Laakso et al. (2018)	FoE	Study relationships between electrophysiological measurements and E-fields in brain to locate the TMS activation site.	19	FDI MTs (active & resting) recorded by anteromedially oriented monophasic TMS across multiple M1 sites; calculated E-fields best explained coil-location-dependent MT variations.	E-field strength in the hand knob varied consistently with MT changes across coil positions (*R*^2^ = 0.69), localizing the activation site to the ventral and lateral hand knob, with substantial interindividual variability in both MTs and computed E-fields.	These findings demonstrate that combining TMS with individualized E-field modeling enables high-resolution functional mapping of the motor cortex by linking scalp coil position to subject-specific cortical activation sites.
A de Goede et al. (2018)	FoE	Examined effects of small coil positioning errors on TMS outcomes. Compare function-guided vs. robot-guided hotspot localization accuracy.	Single pulse study: 8 Paired pulse study: 10	Single-pulse and paired-pulse TMS applied at PA45° from midline, with robotic optical tracking. Coil shifts of 2 mm & 5 mm in 4 directions from the hotspot and ±10° orientation changes (PA35° and PA55°) tested while tilt remained constant.	Group-level MEP, TEPs, and LICI unaffected by positional shifts or ±10° orientation changes, individual variability was notable. Original hotspot never produced largest MEP; several participants showed greater responses at nearby sites.	Group analyses may mask individual sensitivity to coil placement & orientation deviations, suggesting that while PA45° is a reasonable M1 default, optimal parameters could vary across subjects and may require precise navigation.
Souza et al. (2018)	FoE	Assess how coil orientation affects on MEP & spatial centroid in APB by 8 orientations; compare monopolar, bipolar, & high-density EMG recordings.	13	Biphasic TMS was applied at 8 orientations (PA0°–315°, 45° increments) at 120% MT, with 10 pulses per orientation; EMG was recorded via simulated monopolar, bipolar, and a 61-electrode high-density grid.	Peak MEPs at PA45° (largest, ~2 × most orientations) and PA90°, PA135° and PA315° (smallest responses, ~1/6 of PA45°). Conventional electrodes detected peaks at optimal orientations, only the high-density grid reliably captured amplitude differences across non-optimal orientations.	Coil orientation strongly modulates MEP amplitude without shifting the spatial activation center, ~45° maximize responses by aligning E-field perpendicular to the central sulcus, supporting the anatomical basis of the classic 45° rule.
Weise et al. (2020)	FoE	Develop & validate method for determining individually stimulated cortical site in M1 by integrating E-field modeling & physiological MEP data.	Exp. 1, 15 Exp. 2, 3 (from experiment 1)	Exp.1: FEM E-field simulations combined with MEP data using a focal cortical origin model; MEPs recorded from right FDI at PA45°. Exp.2: Multiple coil positions and orientations (−PA90° to +PA90°) across 20 conditions to reduce E-field correlation and enhance localization precision.	Re-optimizing coil position and orientation to maximize the modeled E-field at the predicted hotspot yielded PA-45° orientations and lowest RMTs; reducing E-field correlation across configurations improved localization capability.	Combining subject-specific E-field modeling with physiological data identifies focal cortical activation near sulcal walls, advancing beyond center-of-gravity mapping strategies.
Hand et al. (2020)	FoE	Determine how coil current direction and muscle activation state influence corticospinal and intracortical excitability of a lower-limb muscle.	21	RMT, AMT, single-pulse MEP amplitude, and paired-pulse protocols (SICI, SICF, LICI) assessed for tibialis anterior under PA and ML orientations in resting and active conditions.	Mediolateral (ML) orientation required lower intensity than PA; at rest, ML produced stronger SICI and weaker SICF, while during activation ML showed reduced inhibition and enhanced facilitation relative to PA.	Lower-limb M1 follows different orientation rules than hand M1, with ML more effectively engaging corticospinal pathways; coil direction and muscle state interact to dynamically modulate intracortical excitability.
Tervo et al. (2020)	FoE	Develop and validate an automated closed-loop TMS search method (KG-BOOST) to identify optimal M1 hotspot and coil orientation.	5	Multi-locus TMS with two overlapping FoE coils; KG-BOOST compared against a dense 31-pulse grid search and manual hotspot hunting across three placements and orientations spanning ±90°.	KG-BOOST identified optimal hotspot and ~PA45° orientation in ~18 pulses (~1.5 min), with localization errors of 1.4–2.1 mm and orientation errors of 5.1–7.5°, using ~40% fewer pulses than grid search.	Bayesian optimization efficiently identifies optimal stimulation location and orientation with accuracy comparable to dense grid search while requiring substantially fewer pulses.
Gomez-Tames et al. (2021)	FoE	Quantify E-field thresholds for perception and pain during single-pulse TMS of Broca’s area and M1 to inform safety guidelines.	12	MRI-based individualized head models used; single-pulse TMS delivered at 8 coil orientations by staircase to determine perception/pain thresholds; FEM simulations compute induced E-fields in skin and muscle coupled with a conductance-based axonal model.	Broca’s area showed lower, orientation-dependent thresholds (pain: 85–104 V/m skin; perception: 46–53 V/m skin) versus higher, orientation-independent thresholds for M1; pain thresholds were ~twice perception thresholds.	Lower thresholds in Broca’s area reflect activation of thicker muscle fibers, while M1 thresholds reflect superficial Aδ fiber stimulation, providing quantitative dosimetric data for TMS safety standards.
Tani et al. (2021)	FoE	Determine whether coil orientation modulates scalp pain during single-pulse TMS of BA compared with M1.	15 each Total = 30	Exp.1: Pain thresholds measured across 7 orientations (−PA90° to +PA90°) using an adaptive staircase for both BA and M1. Exp.2: BA only at 110% RMT comparing −PA30° and PA60° orientations (10 trials each).	Pain thresholds varied significantly with orientation in BA but not M1; the PA60° (downward) orientation produced the highest pain threshold and least discomfort, confirmed in Exp.2.	Orientation can be optimized not only for cortical targeting but also to improve tolerability and minimize TMS-related discomfort, particularly for BA stimulation.
Casula et al. (2022)	FoE	Determine the effect of variable coil orientations and stimulation intensities on LMFP, TEP and TRSP	16	FEM with Single-pulse TMS delivered via a FOE coil at PA0°, 90°, 180°, and 270°at 100–140% RMT; EEG and EMG from contralateral FDI were recorded.	Early EEG responses (10–70 ms) were strongest at PA0° and PA90° coil orientations, particularly at 140% RMT. Later responses (70–200 ms) increased significantly only at 0°.	PA0° providing the most robust and selective targeting, whereas PA90° increases potency but reduces focality.
Becker et al. (2025)	FoE	Evaluate the effects of multiple coil directions and stimulation locations on lower limb intracortical excitability during TMS motor mapping.	11	Navigated TMS mapping of tibialis anterior across 6 cortical locations and 8 coil orientations (PA0°–315°, 45° increments) at 100% RMT using surface EMG.	Highest MEPs at orientations perpendicular (PA90°) and dorsolateral (PA135°) to the midline; lowest at inverse-orthogonal orientations (PA225°–315°).	Stimulation perpendicular to the midline (~PA90°) most effectively activates lower-limb motor cortex, confirming strong orientation-dependent modulation of tibialis anterior responses.

C^3^, Cortical Column Cosine; MEP, Motor Evoked Potential; RMT, Resting Motor Threshold; MT, Motor Threshold; APB, Abductor Pollicis Brevis; FDI, First Dorsal Interosseous; TEP, TMS-Evoked Potential; LICI, Long-Interval Intracortical Inhibition; EMG, electromyography; AMT, Active Motor Threshold; SICI, Short-Interval Intracortical Inhibition; SICF, Short-interval Intracortical Facilitation; ML, Mediolateral; KG-BOOST, Knowledge Gradient Bayesian Optimization of Stimulation Targeting; Aδ fiber, A-delta Fiber; BA, Broca’s Area.

#### Experimental validation of model-guided optimization

Two experimental studies directly tested whether computationally guided optimization of coil position and orientation improves stimulation outcomes relative to conventional approaches:

The first study by Weise et al. (2020) combined MEP recordings at multiple coil positions with individual FEM E-field modeling, identifying sharply bounded cortical areas at the gyral crowns and upper sulcal walls of the motor hand area as the origin of MEPs. The magnitude of the tangential E-field component and the overall E-field magnitude were identified as the quantities most relevant to observed MEP amplitude. Furthermore, the optimal coil orientation predicted to maximize E-field and minimize resting motor thresholds was close to the PA45° reference (Weise et al., 2020). Thus, the study by Weise et al. confirms that 45° is a well-founded starting point that can be further individualized with relatively modest computational effort. In addition, the second study by Tervo et al. (2020) evaluated a closed-loop Bayesian optimization algorithm (KG-BOOST) implemented on a multi-locus TMS system to automate simultaneous search for optimal stimulation location and orientation in the FDI representation and identified that optimal orientations consistently fell within approximately 5–7° of PA45°, but with individual variation spanning several tens of degrees, a range clinically meaningful and undetectable by fixed-angle protocols, thus further pointing towards the importance of adjusting for individual variations in optimizing coil orientation.

Similarly, a systematic review of 132 motor-mapping studies by Sondergaard et al. (2021) corroborated the clinical utility of anatomy-guided targeting, concluding that sulcus-aligned orientation improved spatial precision over fixed-angle protocols, while a PA45° default remained adequate when neuro-navigation was unavailable. Collectively, these findings support a tiered approach, PA45° as the practical default in resource-limited settings, and anatomy-guided optimization as the standard when precision is the priority.

#### Studies at the DLPFC

Three studies met the eligibility criteria for the DLPFC: two experimental studies (Thomson et al., 2013; Yang et al., 2025) and one mixed computational and experimental E-field modeling study (Wu et al., 2025). Of these, only Thomson et al. (2013) manipulated coil orientation as an independent variable, demonstrating that hemodynamic responses measured with near-infrared spectroscopy were greatest at approximately PA45°. Wu et al. (2025) showed that functional DLPFC targets exhibited substantially greater spatial dispersion than anatomical targets and that orientation strongly influenced E-field distribution, suggesting that DLPFC targeting is sensitive to angular variation. Yang et al. delivered stimulation at a single fixed mediolateral orientation and did not vary orientation; it is included as contextual evidence that DLPFC stimulation propagates to modulate downstream SMA and M1 activity, and does not contribute to the question of optimal orientation. Together these findings suggest that DLPFC targeting may be sensitive to orientation, but the evidence is insufficient to specify an optimal angle (Table 6).

**Table 6:** Studies examining coil orientation effects in the dorsolateral prefrontal cortex (DLPFC).

Author & Year	Type of Coil Used	Study Design	Aim	Subjects	Methods	Results	Conclusion
Thomson et al. (2013)	FoE	Experimental (orientation manipulated)	Examine whether prefrontal hemodynamic responses (HbO) depend on coil orientation, hypothesize that PA45° produce greater HbO increases.	Study 1: 12 Study 2: 8 (3 from previous)	Single pulses and 1 Hz rTMS trains delivered over the left prefrontal cortex, while bilateral HbO responses were recorded using near-infrared spectroscopy (NIRS) across three orientations (PA45°, PA135°, and PA225°) counterclockwise from the midline in two studies.	Largest HbO changes occurred at PA45° for both single-pulse and rTMS; short rTMS at PA45° greater HbO increases, while PA135° and PA225° yielded comparatively minor effects.	Prefrontal rTMS effects on HbO are orientation-dependent, with PA45° producing superior hemodynamic responses, supporting its preferential use in prefrontal stimulation paradigms.
Wu et al. (2025)	FoE	Computational and Experimental Combined	Evaluate how TMS-induced E-field magnitude differ when left DLPFC is targeted by functional vs. anatomical localization in Alzheimer’s patients & healthy controls.	30	Functional and anatomical DLPFC targets identified; coil initially oriented posterolaterally at 45° then rotated in 30° increments (0°–150°); E-field magnitude and normal component (E⊥) computed using SimNIBS within a 10 mm region of interest.	Functional targets showed 2.63-fold greater spatial dispersion than anatomical targets; aligning coil handle E⊥ to anatomical axis increased at the functional target, producing ~70% stronger stimulation relative to surrounding regions.	Orienting the coil perpendicular to the functional–anatomical axis optimally enhances focal activation while minimizing off-target excitation, representing a superior strategy to the conventional 45° approach,
Yang et al. (2025)	FoE	Experimental (contextual; orientation held fixed)	Investigate whether online 10 Hz rTMS over left DLPFC during motor preparation modulates anticipatory postural adjustments (APAs).	17	Rapid arm-raising task performed during real or sham rTMS over left DLPFC (F3) using a mediolaterally oriented FoE coil at 110% RMT; fNIRS measured HbO in DLPFC, SMA, and M1; surface EMG recorded lumbar multifidus latency and amplitude as the APA index.	Active rTMS reduced HbO in DLPFC, SMA, and M1 versus sham; APA latency shortened, and lumbar multifidus amplitude decreased, indicating more efficient anticipatory postural control mediated through M1.	DLPFC-driven attentional resources modulate M1 output, and targeted prefrontal stimulation can fine-tune motor-cortical control of postural adjustments even with a standard mediolateral coil orientation.

DLPFC, Dorsolateral Prefrontal Cortex; HbO, oxygenated hemoglobin concentration; rTMS, Repetitive TMS; NIRS, Near-Infrared Spectroscopy; fNIRS, Functional Near-Infrared Spectroscopy.

### Optimal coil orientation from human experimental studies at M1 and DLPFC in pain-related studies

#### Pain modulation and analgesic rTMS

Before considering the available evidence, it is important to distinguish the physiological readout used throughout the M1 and DLPFC sections above from the mechanisms that underlie analgesia. MEP amplitude and RMT reflect the excitability of Layer V corticospinal pyramidal neurons and their descending projections to spinal motor circuits. In contrast, rTMS-induced analgesia is thought to arise from top-down modulation of distributed cortico-subcortical and cortico-thalamic pain networks. At M1, projections to the thalamus, periaqueductal grey, and rostroventromedial medulla are thought to engage endogenous descending inhibitory control, while connections to the anterior cingulate and insular cortices act on the affective–motivational rather than the sensory–discriminative dimension of pain (Leung et al., 2020; Galhardoni et al., 2015). At the DLPFC, the proposed route differs: stimulation is thought to modulate the cognitive and affective appraisal of pain through prefrontal–limbic connectivity, consistent with its shared use as a target in depression and in pain (Leung et al., 2020). These are anatomically and functionally distinct systems from the corticospinal projection that MEPs measure. The neurons whose output MEPs index are defined by their descending motor projection, whereas the populations implicated in analgesia are defined by their connectivity to subcortical and limbic structures, and the two may in principle be optimally driven by different field orientations. A coil orientation that maximizes corticospinal MEP output is therefore not guaranteed to optimally engage the networks responsible for pain relief. Accordingly, MEP and RMT should be interpreted as surrogate markers of local cortical excitability rather than direct proxies for analgesic efficacy, and any orientation-dependent claims about pain outcomes require corroboration with clinical pain measures rather than motor physiology alone.

Against the backdrop of well-characterized orientation effects in the motor cortex, the question of whether coil orientation matters for analgesic outcomes is clinically pressing, but based on current evidence, largely unanswered. Of all studies identified across the seven databases covering 1992–2025, only Tani et al. (2021) examined pain outcomes at all. However, no study identified by this review directly compared different coil orientations with respect to therapeutic analgesic outcomes in a clinical pain population. This is a negative finding, and it is the central result of the third review objective.

Tani et al. (2021) measured pain thresholds with single-pulse TMS over two cortical targets: left Broca’s area (BA 44 and 45) and the M1 hand knob at seven coil orientations (−90° to +90° in 30° increments relative to the PA0° and PA45° for the left Broca’s area and M1, respectively). Pain thresholds at M1 were not significantly affected by the tested coil orientation variations. However, at Broca’s area, coil orientation significantly modulated pain thresholds; thresholds were significantly lower at PA30° relative to the 60° downward (PA300° in left PA configuration) condition, and subjective pain intensity at 110% RMT was significantly stronger in the −30° condition. Critically, this study targeted M1 and Broca’s area rather than the DLPFC or other pain-modulating networks, meaning that orientation-dependent effects at the primary clinical targets for therapeutic pain rTMS remain entirely uncharacterized. Overall, these findings represent the entirety of the primary experimental literature on orientation-dependent analgesic TMS effects, and the absence of further studies is itself a substantive finding of this review.

The broader rTMS literature provides context but cannot fill this gap as coil orientation affects analgesic outcomes. Galhardoni et al. (2015) reviewed 33 double-blind, controlled trials and found that high-frequency rTMS applied to M1 with PA-directed currents, typically near PA45°, produced clinically meaningful pain reduction, particularly in neuropathic pain syndromes. Coil orientation was not manipulated as an independent variable in any of these trials, it was held fixed as part of a standardized protocol. Whether the analgesic benefit arose specifically from the PA45°, or whether alternative orientations might produce equivalent or superior effects, remains unknown. A further complication is that stimulation discomfort at the scalp, which is influenced by orientation through differential activation of peripheral nociceptors and muscle fibers, as demonstrated by Tani et al. (2021) and modeled by Gomez-Tames et al. (2021), may not correspond to cortical excitability effects, meaning the angle minimizing discomfort and the angle maximizing cortical analgesic engagement may not coincide. The analgesic mechanism of rTMS likely involves distributed networks extending well beyond the focal motor representations captured by MEPs, yet orientation has never been treated as a systematically optimized variable in any published pain trial (Table 7).

**Table 7:** Review articles examining coil orientation and stimulation parameters in motor cortex TMS.

Author & year	Aim	Methods	Results	Conclusion
Siebner et al. (2009)	Explain the neurophysiological mechanisms by which TMS disrupts cortical activity and clarify implications for cognitive neuroscience studies using TMS as a “virtual lesion” tool, focusing on current direction, intensity, cortical state, and coil orientation.	Single-pulse and brief high-frequency TMS delivered over motor, visual, and association cortices using a FoE coil; behavior, MEPs, and brain activity recorded; paired-pulse protocols assessed interhemispheric interactions; coil orientation was rotated to modulate axonal recruitment.	Current perpendicular to the central sulcus (~PA90°) maximized M1 effects; suprathreshold pulses produced synchronized bursts followed by GABAergic inhibition, while subthreshold pulses introduced neural noise that could impair or enhance perception; effects propagated to connected regions and were modulated by cortical state and orientation.	TMS effects are intensity-dependent and highly sensitive to coil orientation and induced current direction, which together determine focal cortical activation, functional silencing, and network-level propagation.
Galhardoni et al. (2015)	Review analgesic effects of rTMS in chronic pain across syndromes and stimulation parameters.	Systematic review of 33 randomized, double-blind trials (1996–2014; >10 participants) evaluating rTMS protocols (frequency, intensity, orientation) over M1.	High-frequency (≥10 Hz) PA-oriented M1 rTMS (~1,000 pulses/session) produced ≥30% pain reduction in ~50% of patients, particularly neuropathic pain (20–65% response), associated with increased pain thresholds and endogenous opioid release.	Repeated high frequency rTMS over M1 demonstrates consistent analgesic potential across multiple chronic pain syndromes, especially neuropathic pain syndromes, especially neuropathic pain.
Sondergaard et al. (2021)	Synthesize 132 TMS motor-mapping studies to determine how six controllable parameters, including coil orientation, influence mapping outcomes and test–retest reliability.	Compared passive vs. active motor states, fixed PA45° vs. sulcus-aligned orientations, stimulation intensities relative to RMT, stimulator systems, grid designs, and muscle selection across published studies.	Passive mapping showed moderate-to-high reliability; active mapping increased map area; sulcus-aligned orientation improved spatial precision over fixed 45°; ~110% RMT balanced focality and reliability; FDI and APB yielded the most consistent maps.	Fixed PA45° is a practical reproducible standard without neuronavigation, but MRI-guided sulcus-aligned approaches provide superior precision when resources permit.

GABAergic, Gamma-Aminobutyric Acid-ergic (relating to neurons/synapses that use GABA as their neurotransmitter).

#### Coil orientation in the dorsolateral prefrontal cortex

The DLPFC, a widely used treatment target for depression and pain, presents a similar fundamental challenge with no physiological biomarker to guide real-time optimization of coil orientation. In the absence of an MEP-like electrophysiological readout, most clinical DLPFC protocols default to the PA45° orientation derived from motor cortex research, a pragmatic choice for which direct supporting evidence at DLPFC is limited.

Wu et al. (2025) questioned the inherent shortfalls of this default coil orientation and demonstrated with computational modeling and two groups of subjects containing Alzheimer’s Disease and healthy controls that functional targeting showed greater spatial dispersion compared to anatomical targeting. Significant E-field differences were observed between the functional target and adjacent anatomical regions when the coil was positioned over the functional target in both groups. Optimal coil orientation exhibited directional specificity: parallel alignment with the LOI (defined as the line between functional and anatomical targets) E-field produced higher field intensity in the functional target compared to the anatomical target (AD patients: *p <* 0.001; HCs: *p* = 0.052), while perpendicular orientation maintained functional stability with reduced anatomical interference (both groups: *p <* 0.001), suggesting that at the DLPFC, coil orientation variations may result in significant targeting consequences.

Experimental evidence in healthy participants confirms this concern. Hemodynamic responses based on blood oxygenation measurements during rTMS at the prefrontal cortex with multiple orientations were largest at PA45° orientation, and the magnitude of orientation-dependent differences was comparable to that seen at M1 (Thomson et al., 2013), suggesting the equal importance of coil orientation in inducing physiological responses in both targets.

In addition, DLPFC stimulation has been shown to propagate through cortico-cortical pathways to modulate downstream SMA and M1 activity (Yang et al., 2025), these findings establish that suboptimal orientation selection at the DLPFC has consequences not only for prefrontal engagement but for the broader cortical networks these protocols aim to modulate, even though this study did not vary orientation. On the basis of three studies, one of which held orientation fixed, the 45° convention is best regarded as an untested default at the DLPFC rather than a validated optimum. Whether individualized, anatomy-guided orientation improves prefrontal engagement is a testable hypothesis that the current literature does not answer.

## Discussion

The evidence reviewed here converges on a consistent conclusion: that TMS coil orientation is not a universal constant, but a geometry-dependent variable anchored to local sulcal anatomy. Early motor-mapping experiments established the PA45° orientation as a reliable default by demonstrating that it consistently produced the strongest corticospinal responses in the hand representation of M1 (Brasil-Neto et al., 1992; Mills et al., 1992), and computational modeling confirmed that this reflects the angle maximizing E-field alignment with the anterior sulcal wall (Goodwin and Butson, 2015; Janssen et al., 2015). The convention endures because it is grounded in anatomy, not arbitrary.

It is also worth noting that “optimal orientation” is not a single outcome across the studies reviewed here. Computational studies typically optimize E-field magnitude or a specific field component, motor experimental studies optimize MEP amplitude or minimize resting motor threshold; the DLPFC study by Thomson et al. (2013) optimized hemodynamic response; and clinical trials, where orientation was fixed rather than varied, report pain or mood outcomes without reference to any of the above. These endpoints are related but not interchangeable, and a larger modeled E-field does not necessarily correspond to a larger physiological response or better therapeutic outcome. Where possible, we have specified which outcome was optimized in each cited study; this heterogeneity should be kept in mind when comparing conclusions across the M1, DLPFC, and pain-related sections.

Simultaneously, this mechanistic insight could also predict when and why the PA45° convention works for M1 and it could fail elsewhere. If optimal orientation is determined by local sulcal geometry, then any deviation from the stereotyped folding pattern of the M1 hand area, whether due to individual variation, a different motor representation, or a non-motor target, can produce a corresponding deviation from the optimal angle. In fact, the current volume of evidence supports the notion that optimal coil orientation for M1 can vary by approximately ±30° across individuals (Goodwin and Butson, 2015), and the angle variation range can be far greater for other cortical regions such as the DLPFC, lower-limb motor cortex, and pre-SMA, where the cortical geometry differs fundamentally from the hand knob (Casula et al., 2022; Lee et al., 2018). Thus, rather than treating these as exceptions of the 45° rule, they should be understood as confirmation of the underlying principle that matching the field to the sulcal wall is general, even when the resulting angle is not 45°. In the context of pain modulation, coil orientations optimizing motor responses may not be the same as those optimizing analgesia, and critically this has been sparsely tested. This has been one of the major gaps in the existing literature and a limitation of the published clinical trial of rTMS for pain, as whether the orientation used was optimal, suboptimal, or irrelevant to the analgesic mechanism. The only study that manipulated orientation in any pain-related context examined discomfort caused by the stimulus itself, and found orientation-dependent effects at Broca’s area but not at M1. None of the literature assessed had manipulated orientation with respect to relief of an underlying pain condition. The foundational question of this review therefore remains open, and it is open in a specific and answerable way rather than merely under-researched.

Comparisons between computational studies require care, because quantitative predictions depend on modeling choices that vary between groups and are not always reported. Four assumptions could matter significantly. First, tissue conductivity values are assigned from published reference ranges that vary by up to a factor of two between sources, and predicted field magnitudes scale directly with them. Second, most head models treat tissue as isotropic, whereas white matter is anisotropic; incorporating diffusion-derived anisotropy could alter both predicted field magnitude and the location of the predicted activation site, which is one reason tract-targeting studies such as Nummenmaa et al. (2014) report optima that differ from studies modeling gray-matter field magnitude alone. Third, segmentation resolution can determine how faithfully sulcal geometry is represented, and because optimal orientation is defined by sulcal wall angle, segmentation error may propagate directly into orientation error, Makarov et al. (2021) found segmentation accuracy to be the primary determinant of optimization performance. Fourth, models differ in whether they stop at the field distribution or continue to a neuronal activation criterion; those incorporating explicit axon models, such as Abbasi et al. (2024) and Laakso et al. (2025), can predict activation thresholds and current-direction asymmetries that field-magnitude models cannot, and their optima are not directly comparable to field-magnitude optima. These differences explain much of the apparent variation between the computational studies in Table 4, and they mean that convergence on the qualitative conclusion, that optimal orientation follows local geometry, is more informative than agreement on any specific angle.

For DLPFC stimulation, the available computational and experimental findings are consistent with the possibility that MRI-guided, sulcus-perpendicular orientation would outperform the default PA45° approach, particularly given the absence of real-time physiological feedback in prefrontal protocols. On the basis of three studies, only one of which manipulated orientation, this remains a hypothesis requiring prospective validation rather than a supported recommendation.

The finding that optimal orientation is anatomy-dependent could be deemed useful if it can be acted on in settings that lack neuro-navigation or E-field modeling software, and translating it does not require universal adoption of individualized modeling. A tiered approach is more immediately feasible. At the first tier, requiring no additional equipment, clinicians can apply the target-specific heuristic angles summarized in Table 8 rather than defaulting to PA45° for every target: approximately PA45° for the M1 hand representation, PA0° for the pre-supplementary motor area, and PA90° or PA270° for lower-limb motor cortex. Applying the hand-area convention to a lower-limb target is not a neutral default but a predictable and avoidable error. At the second tier, requiring only existing TMS hardware, the orientation producing the lowest resting motor threshold or the largest MEP can be identified empirically at the start of treatment by testing a small number of angles around the first-tier heuristic; this adds minutes rather than sessions, but is not applicable to non-motor targets such as the DLPFC, which is precisely why prefrontal protocols currently default to a motor-derived angle. At the third tier, centers with access to structural MRI and E-field modeling software can compute subject-specific optimal orientation, which is most valuable where inter-individual anatomical variability is greatest: non-hand motor targets, midline targets, and the DLPFC.

**Table 8:** Tiered clinical workflow for selecting coil orientation, with target-specific heuristic angles.

Target	Heuristic angle	Muscle/region	Source
M1 hand—FDI	PA35.5° (L)/PA30.1° (R)	First dorsal interosseous	Bashir et al. (2013)
M1 hand—APB	PA15.6° (L) / PA9.0° (R)	Abductor pollicis brevis	Bashir et al. (2013)
M1 hand—ADM	PA32.4° (L) / PA32.2° (R)	Abductor digiti minimi	Bashir et al. (2013)
M1 lower limb	PA90° / PA270°	Tibialis anterior (foot dorsiflexion)	Hand et al. (2020) and Becker et al. (2025)
Pre-SMA	PA0°	N/A (non-motor target)	Casula et al. (2022)
DLPFC	~PA45° (default; limited evidence)	N/A (non-motor target)	Thomson et al. (2013)

Each tier is defined by the equipment it requires rather than by its precision in principle. Tier 1 requires no additional equipment and applies target-specific heuristic angles in place of a universal PA45° default. Tier 2 requires only existing TMS hardware and identifies the orientation producing the lowest resting motor threshold or the largest motor evoked potential empirically, by testing a small number of angles around the Tier 1 heuristic; this tier is not applicable to non-motor targets such as the DLPFC, which lack a comparable physiological readout. Tier 3 requires structural MRI and E-field modeling software and computes subject-specific optimal orientation. No tier has been shown to improve clinical outcomes; the framework makes orientation an explicit, reported, and reproducible parameter rather than a demonstrated route to better analgesia.

DLPFC, dorsolateral prefrontal cortex; E-field, electric field; M1, primary motor cortex; MEP, motor evoked potential; MRI, magnetic resonance imaging; PA, posterior–anterior; pre-SMA, pre-supplementary motor area; RMT, resting motor threshold; TMS, transcranial magnetic stimulation.

Whether any tier improves clinical outcomes remains untested. Validation will require pain-specific trials that report coil orientation alongside standardized outcome measures, including Quantitative Sensory Testing (QST), Visual Analog Scale (VAS) or Numeric Rating Scale pain scores, treatment dose-reduction, and side-effect and tolerability profiles, none of which has yet been reported in an orientation-controlled pain trial. Until such trials exist, the framework above should be understood as a way of making orientation an explicit, reported, and reproducible parameter rather than as a demonstrated route to better analgesia.

### Limitations

Several limitations should be considered when interpreting the findings of this review. The most critical limitation, and the most important finding relative to the review’s primary objective, is the lack of studies examining coil orientation as an independent variable in pain-related rTMS. Despite pain modulation being the central clinical motivation for this review, only one study by Tani et al. (2021), directly addressed this question, and it did so in a non-clinical population at cortical sites of M1 and Broca’s area, which are not the primary targets of therapeutic pain rTMS protocols. No study was found that manipulated orientation specifically to optimize analgesic outcomes, examined orientation-dependent effects at the DLPFC in a pain population, or compared PA versus alternative orientations for neuropathic pain treatment. The 33 controlled trials reviewed by Galhardoni et al. (2015) all used a standardized PA orientation without testing alternatives, meaning that the impact of coil orientation on analgesic efficacy in every existing pain trial is confounded and remains a primary unresolved question in the clinical application of rTMS for pain. More broadly, the evidence base is profoundly asymmetric: 24 studies addressed M1, 3 addressed the DLPFC, and of these, only 1 examined orientation in a pain context, meaning the three stated objectives are addressed with very unequal evidentiary depth. Conclusions drawn for the DLPFC, and pain sections carry substantially greater uncertainty than those for M1 and should be interpreted accordingly. Second, most clinical pain trials adopt standard motor-mapping orientations without systematically varying coil angle, making it difficult to determine orientation’s independent contribution to analgesic efficacy. Third, computational modeling studies differ in head-model resolution, tissue conductivity assumptions, and neuronal activation criteria, introducing variability in quantitative predictions even when conceptual conclusions converge.

Scopus was not searched, owing to institutional access restrictions. Given the computational and engineering component of this topic, Scopus may index modeling and dosimetry work, particularly conference proceedings, that the databases searched here would not retrieve. IEEE Xplore was searched and returned no records beyond those already identified, which provides some reassurance that the engineering literature is adequately represented, but it does not fully offset the absence of Scopus.

Risk of bias was formally assessed for the 18 experimental/physiological studies using ROBINS-I V2. Three qualifications apply: First, an abbreviated signaling-question set was used, applying the single most decision-relevant question per domain rather than the full battery, so the assessment is less granular than a complete ROBINS-I application. Second, several judgements rest on information the original publications did not report, most commonly whether orientation testing order was randomized or counterbalanced; unreported information was treated as a source of concern rather than assumed absent, which makes these ratings conservative. Third, no formal instrument was applied to the computational and E-field modeling studies, because none currently exists for this study type. This is a gap in the field rather than a limitation specific to this review, but it means the computational evidence has not been appraised on a basis comparable to the human studies. This review was not prospectively registered. PROSPERO does not accept scoping reviews, and no registration was sought elsewhere; the screening and charting procedures described above were applied consistently but were not specified in a prospectively published protocol.

A further limitation concerns the outcome measures on which this review’s M1 conclusions rest. MEP amplitude and resting motor threshold index corticospinal excitability, not analgesia, and the relationship between the two has not been established. Every orientation recommendation derived from motor physiology in this review is therefore a recommendation about how effectively a given angle activates the corticospinal system, and its relevance to pain outcomes is an assumption rather than a demonstrated relationship. A related limitation concerns how discomfort is reported in the pain literature. Stimulation-site discomfort is influenced by coil orientation, intensity, frequency, target, and individual scalp and muscle anatomy, and the trials summarized here generally report tolerability as an aggregate side-effect measure without attributing it to any specific parameter. It is therefore not possible to determine from the published literature how much of the reported discomfort in therapeutic rTMS is orientation-dependent.

### Future directions

Future research should move beyond treating the PA45° coil orientation as a fixed convention and instead consider coil orientation as a modifiable therapeutic parameter. In pain-focused rTMS protocols, systematically varying coil orientation while holding stimulation frequency and intensity constant would allow researchers to directly evaluate the importance of E-field directionality in analgesic effects. Integrating individualized MRI-based E-field modeling into clinical trial design could further improve stimulation targeting, and optimization algorithms that account for gyral geometry, cortical folding patterns, and patient-specific anatomy may identify orientations that differ from conventional motor-mapping angles but produce improved therapeutic outcomes. Future studies should also examine whether optimal orientations differ across pain phenotypes, such as neuropathic versus nociplastic pain, as the underlying cortical network pathophysiology may differ meaningfully. Incorporating TMS-EEG recordings or functional neuroimaging may clarify whether analgesic effects arise from corticospinal activation mechanisms or from broader distributed pain-modulation networks. For DLPFC stimulation, developing reliable neurophysiological or hemodynamic biomarkers analogous to MEPs would substantially advance orientation optimization in affective and pain disorders.

## Conclusion

While the PA45° coil orientation remains a physiologically justified and practically useful starting configuration for M1 stimulation, both computational and experimental evidence demonstrate that optimal orientation is anatomy-dependent rather than universal. For non-hand motor or non-motor cortical regions, the optimal coil orientation for eliciting meaningful physiological response may deviate far from the conventional default coil orientation angle. For clinical contexts where precision is a priority, particularly DLPFC stimulation and pain modulation protocols, subject-specific E-field modeling and neuronavigation-guided targeting represent promising approaches supported by a growing but still limited evidence base. Systematic evaluation of alternative orientations in future clinical trials will be essential to determine whether individualized optimization translates to improved therapeutic outcomes. Coil orientation is one addressable source of inter-individual variability in current TMS practice, and one that is unusually tractable: unlike cortical anatomy or network connectivity, it can be changed at no cost during any stimulation session. It is not, however, the only such source, and it should not be presented as the dominant one. Target definition, coil-to-cortex distance, stimulation intensity, pulse waveform, frequency, cumulative dose, cortical state, and network connectivity all contribute, and none has been systematically compared against orientation for its relative influence on clinical outcome. Establishing that relative contribution is itself a research priority.
