# Supplementary material for: TMS coil orientation: from computational modeling to experimental evidence and analgesic considerations
Source: Front Neurosci. 2026 Oct 19;20:1938946. doi: 10.3389/fnins.2026.1938946 (PMC13725392; doi:10.3389/fnins.2026.1938946)
Supplement: Supplementary file 1 [file Table_1.docx]

**Supplementary Table S1.** Justifications for each ROBINS-I V2 domain judgement reported in Table 3.

| Study | D1 Confounding | D2 Classification | D3 Selection | D4 Missing data | D5 Outcome measurement | D6 Selective reporting |
| --- | --- | --- | --- | --- | --- | --- |
| Brasil-Neto et al. 1992 | Moderate: fixed clockwise 45° sequence; no counterbalancing reported | Low: orientations physically set and verified across 0–315° | Low: all 10 participants analyzed; no exclusions | Low: complete data; 4 MEPs per orientation | Moderate: amplitudes read from oscilloscope traces by unblinded assessor | Moderate: no prospectively registered protocol |
| Fox et al. 2004 | Moderate: orientation and movement conditions delivered in an order not reported as randomized | Low: coil position and orientation defined relative to the stimulation site | Serious: analyzed sample restricted post hoc to participants with usable activation maps; selection related to the outcome | Low: complete imaging data reported for the analyzed sample | Moderate: automated PET activation extraction, assessors aware of condition | Moderate: no prospectively registered protocol |
| Opitz et al. 2013 | Low*: order of PA45°/PA90° counterbalanced across participants | Low: MR-navigated, <1 mm positioning tolerance | Low: all 5 participants included; no exclusions | Low: complete data | Moderate: automated MEP extraction via custom scripts, unblinded | Moderate: no prospectively registered protocol |
| Richter et al. 2013 | Low*: coil orientations and session order randomized | Low: robotized coil positioning, MRI-confirmed gyral orientation, 1.8 mm accuracy | Moderate: 1/8 participants excluded post hoc for lacking a clear threshold minimum; 2 completed only 1 of 2 sessions owing to intolerance | Low: exclusions documented and justified | Low: double-blind motor-threshold estimation; robot operator alone aware of orientation | Moderate: no prospectively registered protocol; preliminary results previously reported in a conference abstract |
| Bashir et al. 2013 | Moderate: fixed 45° sequence with 5° fine-tuning; no randomization reported | Low: MRI-neuronavigated coil tracking | Low: all 10 participants included; 4 retested for reproducibility | Low: complete data | Moderate: automated peak-to-peak MEP measurement, unblinded | Moderate: no prospectively registered protocol |
| Bungert et al. 2017 | Low*: 9 coil orientations tested in pseudorandomized order | Low: neuronavigated using individual MRI head models | Moderate: 2/11 participants excluded post hoc on the basis of mapping results | Low: complete data for the 9 analyzed participants | Moderate: sigmoid-fit motor-threshold estimation, unblinded | Moderate: no prospectively registered protocol |
| Laakso et al. 2018 | Moderate: order of coil locations not reported as randomized | Low: geodesic-distance coil tracking, MRI-verified | Low: all 19 participants retained; subgroup differed only by planned protocol | Low: complete data | Moderate: adaptive threshold-hunting algorithm, unblinded | Moderate: no prospectively registered protocol |
| de Goede et al. 2018 | Moderate: fixed session order per protocol table; not randomized | Low: robot-guided navigation, 1 mm tracking accuracy | Moderate: 1 participant skipped the 5 mm locations and 1 participant’s orientation was left unchanged; documented and likely unrelated to outcome | Moderate: incomplete data for 2/8 single-pulse participants | Moderate: automated cluster-based amplitude analysis, unblinded | Moderate: no prospectively registered protocol |
| Souza et al. 2018 | Moderate: 8 orientations delivered in 45° steps; sequence not reported as randomized or counterbalanced | Low: orientations physically set at defined 45° increments from PA0° to PA315° | Low: all 13 participants analyzed; no exclusions reported | Low: 10 pulses recorded per orientation per participant | Moderate: MEP amplitude and centroid extracted from EMG without blinding to orientation | Moderate: no prospectively registered protocol; all 8 orientations reported |
| Hand et al. 2020 | Moderate: test blocks randomized between PA and ML orientations, but activation state not counterbalanced | Low: PA and ML orientations defined, hotspot rechecked throughout | Serious: substantial attrition tied to achievability of response: 8/20 could not reach resting MEP threshold and 1 withdrew | Serious: large proportion of intended resting-state data unavailable; final n varies across analyses (20/12/11) | Moderate: automated peak-to-peak MEP measurement, unblinded | Moderate: no prospectively registered protocol |
| Tervo et al. 2020 | Low*: closed-loop algorithm selects orientations adaptively, removing fixed-sequence effects | Low: multi-locus TMS with electronically defined orientation | Low: all 5 participants analyzed | Moderate: adaptive sampling means not all orientations are sampled equally in every participant | Moderate: automated MEP extraction, unblinded | Moderate: no prospectively registered protocol |
| Weise et al. 2020 | Low*: 20 position and orientation conditions distributed to minimize E-field correlation | Low: neuronavigated with individual FEM head models | Moderate: Experiment 2 conducted in 3 participants drawn from Experiment 1 | Moderate: reduced sample in Experiment 2 | Moderate: automated MEP extraction, unblinded | Moderate: no prospectively registered protocol |
| Gomez-Tames et al. 2021 | Moderate: 8 orientations delivered by staircase procedure; order not reported as counterbalanced | Low:MRI-based individualized head models with defined orientations | Low: all 12 participants analyzed | Low: complete threshold data reported | Moderate: perception and pain thresholds are participant-reported and cannot be blinded to orientation | Moderate: no prospectively registered protocol |
| Tani et al. 2021 | Low*: adaptive staircase across 7 orientations; Experiment 2 conditions counterbalanced | Low: orientations defined relative to PA reference at both targets | Low: all participants analyzed in both experiments | Low: complete data | Moderate: pain threshold and subjective intensity are participant-reported; participants cannot be blinded to orientation | Moderate: no prospectively registered protocol |
| Casula et al. 2022 | Low*: orientations and intensities delivered across a balanced factorial design | Low: neuronavigated FOE coil at defined PA0°/90°/180°/270° | Low: all 16 participants analyzed | Low: complete EEG and EMG data | Moderate: TEP and spectral measures extracted by unblinded assessors | Moderate: no prospectively registered protocol |
| Becker et al. 2025 | Serious: fixed clockwise sequence with no randomization; authors themselves note possible cumulative stimulation effects | Low: neuronavigated, grid-guided stimulation | Low: all 11 participants completed; no exclusions | Low: complete data, 240 stimulations per participant | Serious: EMG responses re-evaluated retrospectively; unblinded, with subjective threshold judgement below 50 µV | Moderate: no prospectively registered protocol; hotspot determination not incorporated into the rotation analysis |
| Thomson et al. 2013 | Moderate: three orientations delivered in an order not reported as counterbalanced; two separate studies pooled | Low: orientations defined at PA45°/135°/225° from the midline | Moderate: Study 2 included 3 participants carried over from Study 1 | Low: complete NIRS data reported | Moderate: HbO extraction unblinded to orientation | Moderate: no prospectively registered protocol |
| Yang et al. 2025 | Moderate: active and sham conditions compared at a single fixed orientation; baseline arousal and expectation not controlled | Low: coil orientation fixed and reported (mediolateral) | Moderate: analyzed sample smaller than recruited in some measures | Moderate: fNIRS and EMG channel dropout not fully reported | Moderate: HbO and EMG measures extracted unblinded | Moderate: no prospectively registered protocol |

***** Domain 1 only: “low risk of bias except for concerns about uncontrolled confounding.”
